# Timing of SMN replacement therapies in mouse models of spinal muscular atrophy: a systematic review and meta-analysis

**DOI:** 10.1093/braincomms/fcae267

**Published:** 2024-08-12

**Authors:** Helena Chaytow, Anna A L Motyl, Yu-Ting Huang, Charis Wong, Gillian L Currie, Zsanett Bahor, Emily Sena, Thomas H Gillingwater

**Affiliations:** Edinburgh Medical School: Biomedical Sciences, University of Edinburgh, Edinburgh EH8 9AG, UK; Euan MacDonald Centre for Motor Neuron Disease, University of Edinburgh, Edinburgh EH16 4SB, UK; Edinburgh Medical School: Biomedical Sciences, University of Edinburgh, Edinburgh EH8 9AG, UK; Euan MacDonald Centre for Motor Neuron Disease, University of Edinburgh, Edinburgh EH16 4SB, UK; Edinburgh Medical School: Biomedical Sciences, University of Edinburgh, Edinburgh EH8 9AG, UK; Euan MacDonald Centre for Motor Neuron Disease, University of Edinburgh, Edinburgh EH16 4SB, UK; Centre for Clinical Brain Sciences, University of Edinburgh, Edinburgh EH16 4SB, UK; Anne Rowling Regenerative Neurology Clinic, University of Edinburgh, Edinburgh EH16 4SB, UK; MRC Clinical Trials Unit, University College London, London WC1V 6LJ, UK; Centre for Clinical Brain Sciences, University of Edinburgh, Edinburgh EH16 4SB, UK; Centre for Clinical Brain Sciences, University of Edinburgh, Edinburgh EH16 4SB, UK; Centre for Clinical Brain Sciences, University of Edinburgh, Edinburgh EH16 4SB, UK; Edinburgh Medical School: Biomedical Sciences, University of Edinburgh, Edinburgh EH8 9AG, UK; Euan MacDonald Centre for Motor Neuron Disease, University of Edinburgh, Edinburgh EH16 4SB, UK

**Keywords:** SMA, systematic review, meta-analysis, pre-clinical, early treatment

## Abstract

Mutations in the *Survival of Motor Neuron 1* gene lead to a loss of survival motor neuron protein in patients with spinal muscular atrophy. Revolutionary advances in gene therapy have led to survival motor neuron-replacement therapies that significantly prolong life expectancy and improve neuromuscular function. However, accumulating evidence suggests that the timing of survival motor neuron-replacement therapies is a critical determinant of success. We performed a systematic review and meta-analysis of all pre-clinical studies testing survival motor neuron replacement therapies in mouse models of spinal muscular atrophy to assess the impact of timing of delivery on therapeutic effectiveness. We incorporated four databases in this pre-registered study (PROSPERO 2020 CRD42020200180): EMBASE, PubMed, Scopus and Web of Science. Inclusion criteria were; primary research article, a measure of survival analysis, use of survival motor neuron mouse model and evaluation of survival motor neuron-targeting therapy. Exclusion criteria included; use of therapies not known to directly target survival motor neuron, genetic manipulations and/or lack of appropriate controls. We screened papers using the SyRF platform. The main outcome we assessed was survival in treated groups compared to untreated groups. We performed meta-analysis of survival using median survival ratio and the random effects model and measured heterogeneity using the *I*^2^ statistic. Subgroup analyses were performed to assess treatment efficacy based on timing of intervention (embryonic delivery, day of birth, postnatal day 2 and postnatal day 3 or later) and treatment type. If detailed in the studies, body weight compared to untreated spinal muscular atrophy models and motor neuron number were included as secondary outcomes for meta-analysis. 3469 studies were initially identified, with 78 ultimately included. Survival motor neuron-replacement therapies significantly affected survival in favour of treatment by a factor of 1.20 (95% CI 1.10–1.30, *P* < 0.001) with high heterogeneity (*I*^2^ = 95%). Timing of treatment was a significant source of heterogeneity (*P* < 0.01), with earlier treatment having a greater impact on survival. When stratified by type of treatment, earlier treatment continued to have the strongest effect with viral vector replacement therapy and antisense oligonucleotide therapy. Secondary outcome measures of body weight and spinal motor neuron counts were also positively associated with early treatment. Earlier delivery of survival motor neuron replacement therapies is therefore a key determinant of treatment efficacy in spinal muscular atrophy.

## Teaser

Chaytow *et al*. conducted a systematic review and meta-analysis for all pre-clinical studies testing therapies that replace levels of survival motor neuron protein in mouse models of spinal muscular atrophy, assessing the impact of treatment timing on effectiveness. Earlier delivery was found to be a key determinant of therapeutic efficacy.

## Graphical Abstract

**Graphical Abstract:**
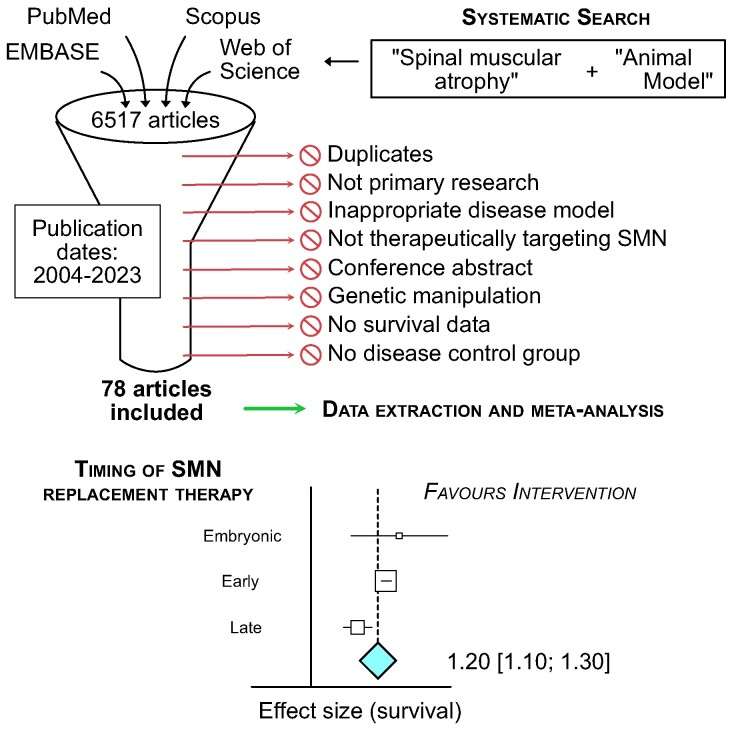

## Introduction

Spinal muscular atrophy (SMA) is a rare monogenic disease (∼1:6000–10 000 live births), which primarily affects lower motor neurons and leads to muscle atrophy.^1,2^ SMA is caused by homozygous mutations in the *Survival of Motor Neuron 1* (*SMN1*) gene, with low levels of the survival motor neuron (SMN) protein (necessary for life) maintained by a copy of near-identical *SMN2* gene.^3^*SMN2* differs from *SMN1* by only a few nucleotides, which leads to a splicing pattern that causes exclusion of exon 7 and only 10% of the full-length SMN protein production.^4,5^ In most cases, SMA is a severe disease with symptoms appearing within six months after birth.^6^

Historically, SMA was a leading genetic cause of infant death worldwide, with patients not routinely surviving past the age of two.^7^ However, notable therapeutic progress has been achieved since 2016, with improved standards of care and the consecutive approvals of three disease-modifying therapies: a splice-modifier antisense oligonucleotide (ASO) targeting *SMN2* for exon 7 inclusion (nusinersen, Biogen), a gene replacement therapy delivering *SMN1* using a modified viral vector (onasemnogene abeparvovec-xioi, Novartis), and another splice-modifier small molecule (risdiplam, Roche).^8^ With these treatments, SMA infants now reach motor milestones previously thought impossible. Nevertheless, some patients respond better than others to these therapies, raising questions about the optimal timing of delivery of these treatments.

With SMN being expressed at high levels during embryonic and prenatal stages of development and symptoms appearing soon after birth, determining the best therapeutic time-window has become a crucial topic in the field.^9^ Results from individual preclinical studies in mouse models of SMA suggest that early treatment is required for maximum therapeutic benefits.^10^ Importantly, emerging data from recent clinical trials suggest that current therapies are, indeed, more efficient when administered in pre-symptomatic SMA Type I patients.^11-13^

Given the widespread availability of SMN-restoring therapies, alongside their high purchase costs, quantitatively reviewing the state of SMA preclinical research is required to identify how best to use these therapies. This will maximize patient benefits and the financial return on investment for the health care provider.^14,15^ For example, a growing number of countries are adding or considering adding SMA to their newborn genetic screening programmes (NBS).^16^ NBS is critical for pre-symptomatic administration of treatments and the SMA community needs reliable evidence to support its adoption. While many individual pre-clinical studies have proposed that there is an early therapeutic time-window for SMA, at the time we registered a protocol for this systematic review, there was no comprehensive and quantitative comparison of all those studies collectively. Despite being the gold-standard for clinical practice, systematic reviews remain atypical in preclinical research.^17^ Here, we aim to address this gap by performing a systematic review and meta-analysis of SMN-targeting therapies in mouse models of SMA. Specifically, we set out to determine the effect of timing of SMN replacement on the primary outcome median survival and the secondary outcomes of body weight and motor neuron count.

## Materials and methods

The CAMARADES group at the University of Edinburgh provided methodological support and guidance.

### Search strategy and selection criteria

This study is a systematic review with meta-analysis. Our protocol was registered in PROSPERO: www.crd.york.ac.uk/prospero/display_record.php? ID=CRD42020200180. Some adjustments to the protocol were made while conducting the data extraction and meta-analysis, which are highlighted in the relevant sections.

We conducted the initial literature search on 22 July 2020. We used EMBASE, PubMed, Scopus and Web of Science (search terms are provided in Supplementary material). This search was then re-run to update our search results on 8 June 2021 using PubMed with the previous search terms, limiting date of publication between 22 July 2020 and 8 June 2021, and on 19 February 2024 limiting date of publication from 2021 to 19 February 2024. We removed duplicates from the library using the automated systematic search deduplication tool: https://camarades.shinyapps.io/RDedup/. Code for the tool is available on: https://github.com/camaradesuk/ASySD.^18^

The de-duplicated library was uploaded into the systematic review facility online platform (SyRF; syrf.org.uk), developed by the CAMARADES group at the University of Edinburgh.^19^ Two researchers independently screened each paper for inclusion (two of H.C., A.M. or Y.-T.H.) with disagreements reconciled by the third reviewer. Initial title-abstract screening removed clinical studies, case studies and reviews as per the protocol. Inclusion criteria were: use of an SMA mouse model and evaluation of an SMN-targeting therapy. Screening for presence of survival data (primary outcome) were left until full-text screening. Full-text screening and data extraction were independently performed by two researchers (two of H.C., A.M. or Y.-T.H.) and collected data were reconciled by the third researcher. Our exclusion criteria were no full text available, no primary outcome reported and no relevant control group. It is important to note that we included all studies presenting survival data and fulfilling our other inclusion/exclusion criteria, without limiting ourselves to those publications comparing different intervention time points. This way, we avoided a bias towards studies performing early intervention and were able to include a broader collection of comparisons, thus increasing the power of our meta-analysis.

### Statistical analysis

We extracted the following data: study design information including experimental groups, type of control and number of animals per group; SMA model; dose, timing, frequency and route of administration (*post hoc* exploration). We included the multiple comparisons (between various experimental groups, routes of administration, doses, etc) described in each paper as individual comparisons. The primary outcome of median survival was read from Kaplan–Meier curves using an onscreen ruler. As SMA mouse models have a lower body weight compared to littermate controls, increased body weight following SMN-targeting treatment is often used as a phenotype to indicate therapeutic efficacy. We therefore recorded body weight as a secondary outcome (on day of maximum body weight recorded for untreated SMA mice, in a deviation from protocol). Protection against the specific motor neuron cell death seen in SMA is another indicator of a successful treatment, and so motor neuron counts were recorded with standard error or standard deviation if available. If there was no primary outcome of survival reported, the paper was excluded. During reconciliation, quantitative data from the two reviewers differing by more than 10% was reconciled by the third reviewer. Timing of SMN replacement therapy was binned into four categories: embryonic treatment (*E*); day of birth (P0/1); second day (P2) and third day or later (P3+). Risk of bias was assessed using modified questions from SYRCLE’s Risk of Bias tool.^20^ We used the SyRF tool to record study information, risk of bias data and primary and secondary outcome measurements from all included articles.

We assessed survival data using median survival ratio as previously described.^21,22^ where median survival was used to calculate the effect size using the following formula:


$$ES_{Rx}=\;\ln\;\left(\frac{\mathrm{median}\;\mathrm{survival}\;\mathrm{in}\;\mathrm{treatment}\;\mathrm{group}}{\mathrm{median}\;\mathrm{survival}\;\mathrm{in}\;\mathrm{control}\;\mathrm{group}}\right)$$

where standard error of the effect size was calculated as the following:


$$SE_{ES}=\;\frac{1}{\sqrt{N_{Rx}+\;N_{C}^{\prime }}}$$

where *N_Rx_* = number of animals in treatment group and *N*′_C_ (true number of control animals) was calculated with the following:


$$N_{C}^{\prime }=\;\frac{N_{C}}{\mathrm{number}\;\mathrm{of}\;\mathrm{treatment}\;\mathrm{groups}\;\mathrm{served}\;\mathrm{by}\;\mathrm{control}\;\mathrm{group}}$$

Forest plots were then generated summarizing treatment effect and compared using random effects meta-analysis with REML estimates of tau. The random-effects model was used due to high heterogeneity. Comparisons were weighted by number of animals in study as a surrogate marker for inverse variance. Heterogeneity was determined using the *I*^2^ statistic. Where more than 25 studies were available per group, data were further analysed by subgroups (i.e. for treatment type). Secondary outcomes were also evaluated using the random effects model. Significance was taken as a Bonferroni-corrected *P*-value of 0.01. Publication bias was assessed using funnel plots with trim and fill analysis and Egger’s regression.

### Role of the funding source

The funders had no input into, or influence on, study design, implementation or interpretation.

## Results

**Figure 1:**
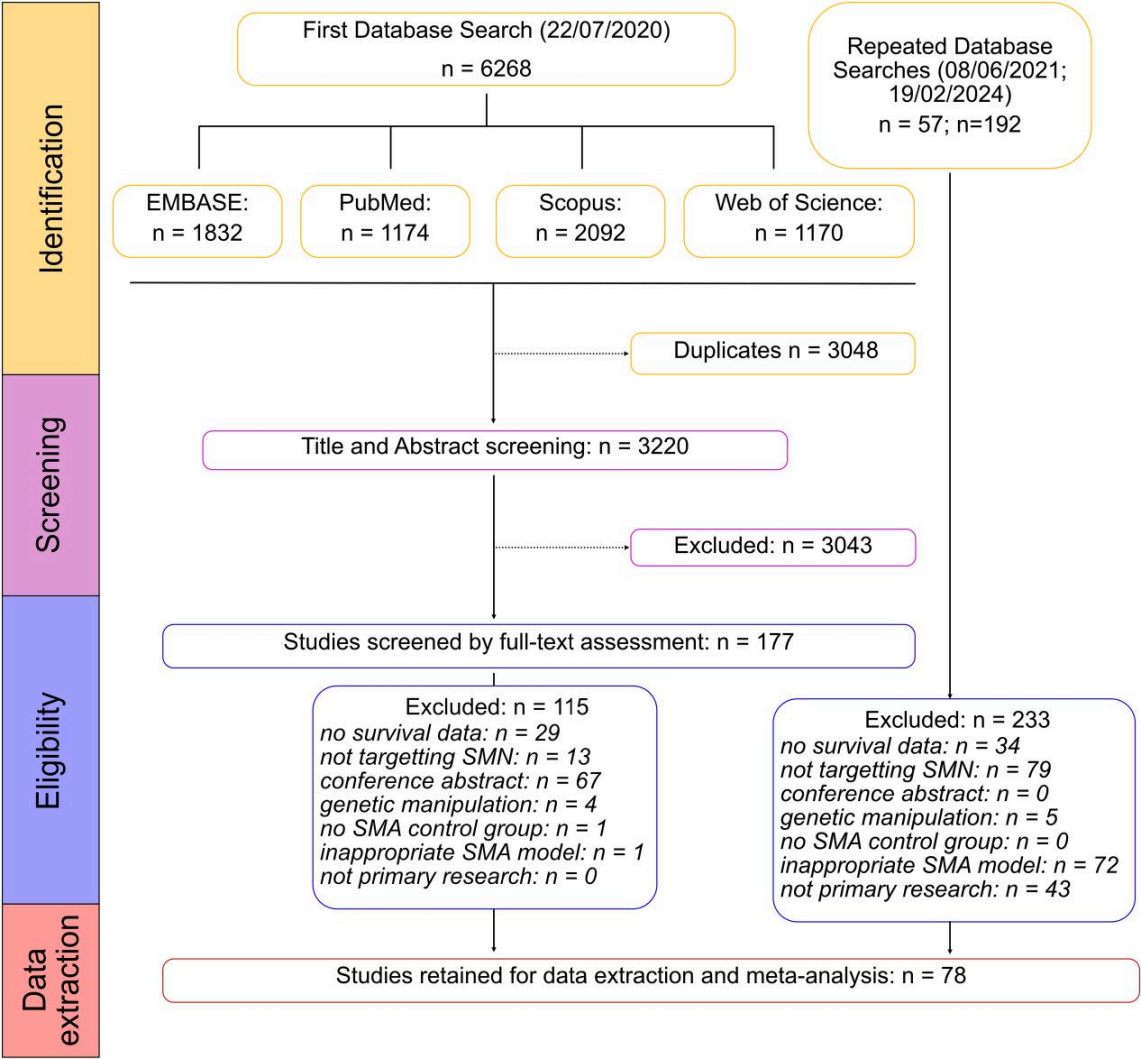
*PRISMA* flow diagram showing the systematic database searches and inclusion/exclusion stages.

Our systematic search identified the following number of articles from each of the four databases: EMBASE (832 articles), PubMed (1174 articles), Scopus (2092 articles) and Web of Science (1170 articles) (Fig. 1). After de-deduplication, the complete dataset included 3220 unique articles. After title-abstract screening we included 177 articles. We then collected the full texts of these articles and screened against our exclusion criteria: 115 articles were excluded in this round, with reasons detailed in Fig. 1. This left 62 articles for data extraction. This search was repeated on 8 June 2023 and 19 February 2024, adding a further 16 articles after full-text assessment. We therefore had a final collection of 78 articles included for data extraction and meta-analysis (Supplementary Table 1).

**Figure 2:**
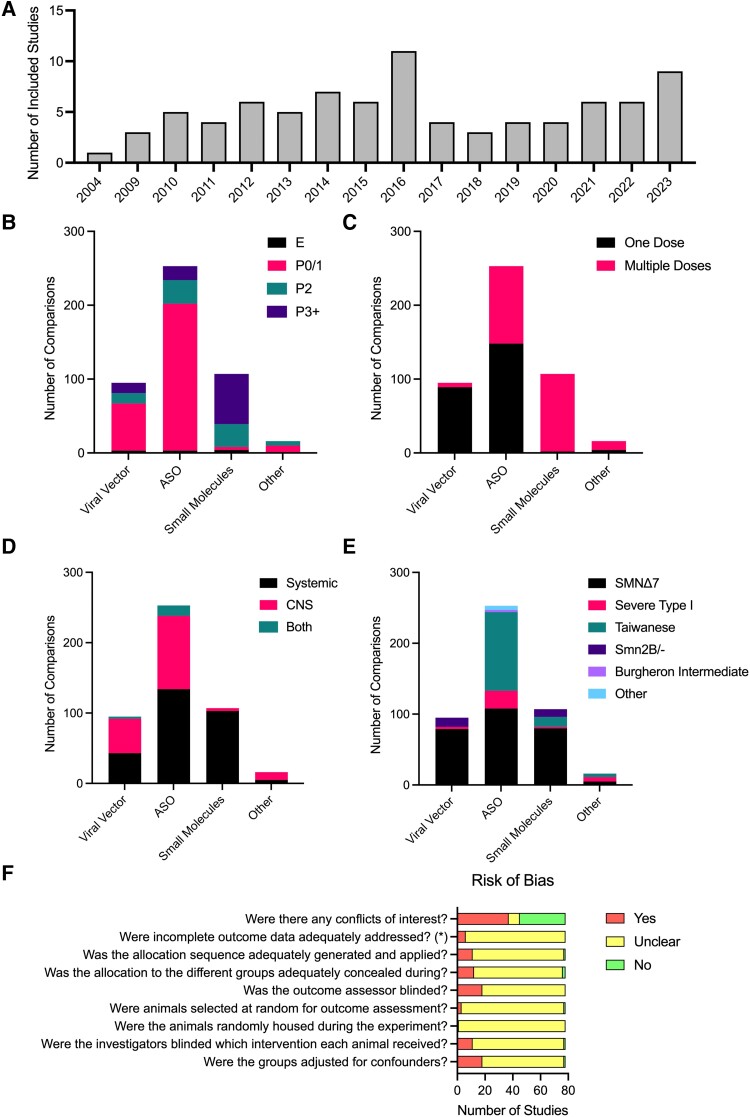
**Characteristics of included studies**. (**A**) Included studies per year of publication. Number of comparisons per therapy type per timing of treatment (**B**), frequency of administration (**C**), route of administration (**D**) and SMA mouse model (**E**). (**F**) Risk of Bias assessment based on the SYRCLE Risk of Bias tool.^20^ (E) Embryonic; P0/1, postnatal day 0 or 1 (day of birth); P2, postnatal day 2; P3+, postnatal day 3 or later. ASO, antisense oligonucleotide; CNS, central nervous system.

The publication dates of included articles spanned from 2004 to 2023 (Fig. 2). Many of the 78 studies included (Supplementary Table 1) describe multiple experimental cohorts, comparing different treatments, doses, routes of administration, etc. Our meta-analysis is thus based on the comparisons from experimental cohort (386 in total), rather than total number of studies. Sixty-five comparisons used viral vector delivery of SMN, 230 comparisons used ASOs, 77 comparisons used small molecules and 14 comparisons used other drugs (*k* values; Fig. 2). The most common timing of treatment was P0/1 (*k* = 244; Fig. 2B). Most comparisons with viral vector delivery of SMN treated mice in a single dose (*k* = 61), although some did investigate multiple dosing of viral vectors (*k* = 4; Fig. 2C). ASOs were either given in one dose (*k* = 120) or over multiple doses (*k* = 110), while small molecule therapies were always given as multiple doses (*k* = 77; Fig. 2C). Viral vectors and ASOs were either delivered systemically (intravenous, intraperitoneal, subcutaneous or oral; *k* = 29 and 116, respectively) or central nervous system (CNS)-directed (intracerebroventricular; *k* = 33 and 103, respectively), while small molecules were predominately delivered systemically (*k* = 75; Fig. 2D). The SMNΔ7 mouse model was the most common (*k* = 212), while the Taiwanese SMA mouse model was also commonly used with ASOs (*k* = 89; Fig. 2E). Note that the Smn2B/- model cannot be used for research directed at the ISS-N1 site, as this sequence is not part of the transgene in these animals.

Determining the reliability of a study is crucial when conducting a systematic review, where the risk of bias assessment for studies included in a review should inform interpretation of the evidence. The SYRCLE Risk of Bias tool^20^ has adapted questions from the Cochrane Risk of Bias tool to be relevant to preclinical animal studies, assessing elements of experimental design such as whether experimenters are blinded to treatment and whether all data have been reported. The majority of studies did not report whether randomization or blinded assessment of outcome were performed (assessed as ‘unclear’ when assessed using the SYRCLE Risk of Bias tool). Most studies reported whether there was a financial conflict of interest, with 31 studies declaring a conflict of interest (Fig. 2F).

**Figure 3:**
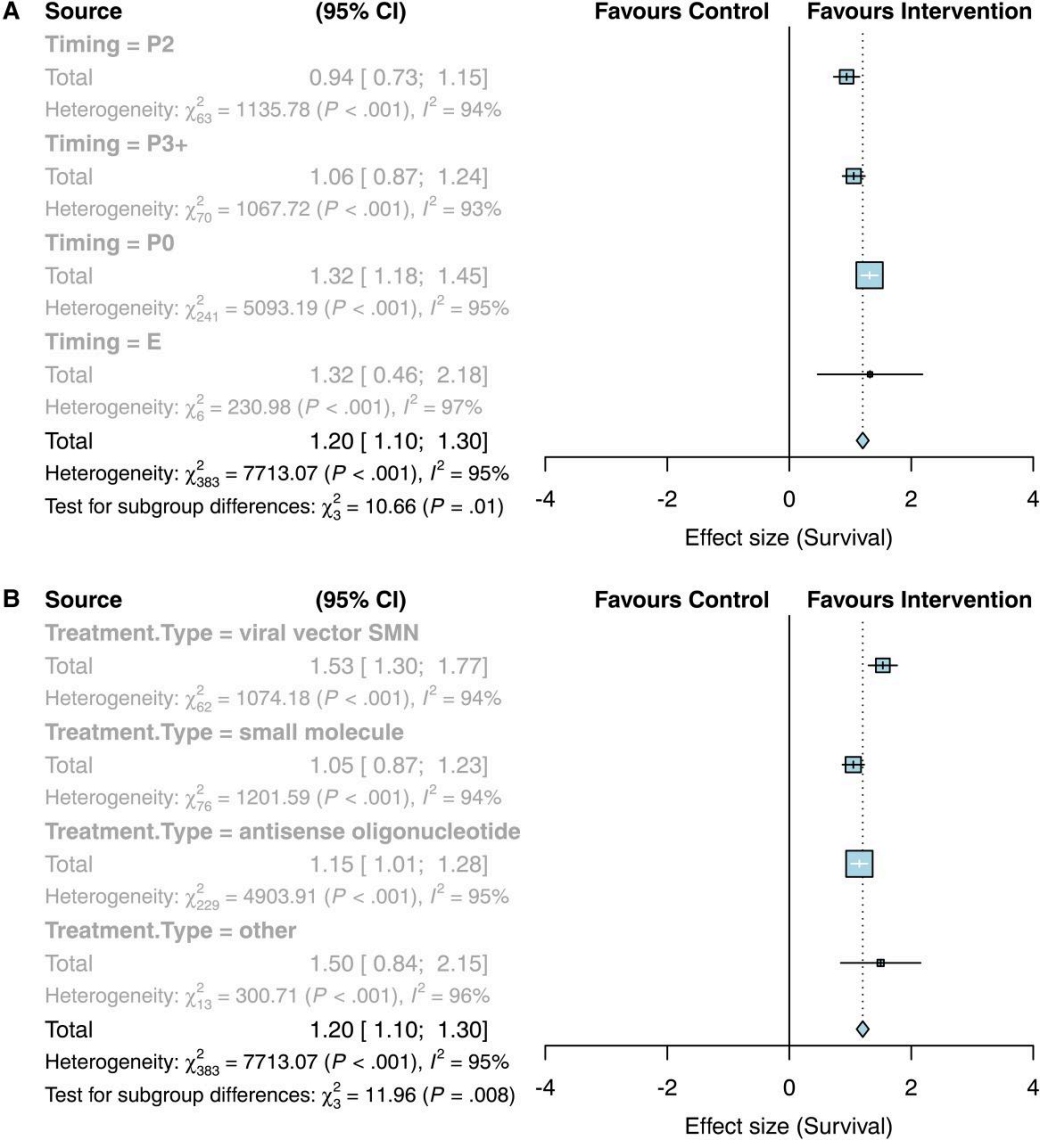
**Forest plots showing a positive effect of SMN replacement therapies on survival, with subgroup analyses per timing and type of treatment**. (**A**) Subgroup per timing. E, embryonic treatment; P0, treatment on day of birth or postnatal day 1; P2, postnatal day 2; P3+, treatment on postnatal day 3 or later. (**B**) Subgroup analysis per treatment type.

The primary outcome measure for this review was survival following SMN replacement therapy. Meta-analysis of survival following SMN replacement therapies showed that there was a significant impact favouring intervention by a factor of 1.20 (95% CI 1.10–1.30, *k* = 386, Fig. 3;Supplementary Fig. 1) and significant heterogeneity (*I*^2^ = 95%; Fig. 3). Subgroup analysis showed that timing of treatment had a significant impact on survival (*P* < 0.01) with the strongest effect at embryonic stage at a factor of 1.32 (95% CI 0.46–2.18; *P* < 0.01) or treatment at P0/1 with a factor of 1.32 (95% CI 1.18–1.45; *P* < 0.01), then treatment at P3 or later at a factor of 1.06 (95% CI 0.87–1.24; *P* < 0.01) and finally treatment at P2 with a factor of 0.94 (95% CI 0.73–1.15; *P* < 0.01) as shown in Fig. 3A. Type of SMN replacement therapy (ASO, viral vector, small molecule or other) was also a significant source of heterogeneity (*P* < 0.01; Fig. 3B). As there were more than 25 studies in the three main groups of therapies, the dataset was stratified according to type of therapy and reanalysed, as pre-specified in our protocol. While the treatment type group ‘other’ had a strong effect on survival (1.50 [0.84–2.15], *P* < 0.001), there were only 14 comparisons in this group and so it did not meet our criteria for sensitivity analysis. These comparisons investigated viral vector delivery of components to enhance *SMN2* splicing (Coady *et al*., Shababi *et al*., Odermatt *et al*., and Donadon *et al*., details in Supplementary Table 1). Although they do not fit into either category of ‘viral vector delivery of SMN’ or ‘ASO’, the strong positive effect of this group emphasizes the successful outcome on survival when targeting SMN replacement.

We therefore repeated our assessment of the effect of SMN replacement therapies on survival with the dataset split according to type of treatment. Various subgroup analyses were performed to determine if they were sources of heterogeneity. Timing of SMN replacement therapy continued to be a significant source of heterogeneity following viral vector therapy and ASO therapy (*P* < 0.01, Table 1), whereas timing was not a significant factor following small molecule therapy (*P* = 0.07). Nevertheless, embryonic and P0/P1 treatment with small molecules showed bigger effect sizes than later treatment. There was a clear trend of increasing effect on survival following earlier treatment for viral vector therapy. Whilst there was no significant effect on survival with embryonic treatment of ASO therapy (0.01 [−0.23–0.25], *P* = 0.46), there was an increased effect on survival following ASO treatment on P0/1 compared to P2 or P3 + treatment (Table 1).

**Table 1:** Summary table of survival statistics after the dataset was split according to type of treatment

	Viral vectors	ASOs	Small molecules
Total effect on survival per treatment type	1.51 [1.28–1.74; *P* < 0.01]	1.12 [0.99–1.26; *P* < 0.01]	1.02 [0.85–1.20; *P* < 0.01]
Timing of treatment			
Subgroup differences?	*P* < 0.01	*P* < 0.01	*P* = 0.07
** ** *E*	2.13 [1.79–2.47; *P* = 0.28; *k* = 2]	0.01 [−0.23–0.25; *P* = 0.46; *k* = 2]	1.68 [0.35–3.01; *P* < 0.01; *k* = 3]
** **P0/1	1.62 [1.35–1.88; *P* < 0.01; *k* = 49]	1.20 [1.04–1.35; *P* < 0.01; *k* = 182]	1.15 [−1.11–3.41; *P* < 0.01; *k* = 33]
** **P2	1.21 [0.46–1.96; *P* < 0.01; *k* = 6]	0.90 [0.53–1.26; *P* < 0.01; *k* = 33]	0.74 [0.55–0.92; *P* < 0.01; *k* = 22]
** **P3+	0.92 [0.37–1.47; *P* < 0.01; *k* = 8]	0.92 [0.57–1.27; *P* < 0.01; *k* = 13]	1.10 [0.87–1.33; *P* < 0.01; *k* = 50]
Frequency of Administration			
Subgroup differences?	*P* = 0.18	*P* < 0.01	N/A
** **One dose	1.47 [1.24–1.71; *P* < 0.01; *k* = 61]	0.80 [0.64–0.97; *P* < 0.01; *k* = 120]	N/A [*k* = 0]
** **Multiple doses	2.13 [1.20–3.06; *P* < 0.01; *k* = 4]	1.48 [1.28–1.68; *P* < 0.01; *k* = 110]	1.02 [0.85–1.20; *P* < 0.01; *k* = 77]
SMA model			
Subgroup differences?	*P* < 0.01	*P* < 0.01	*P* < 0.01
** **Severe Type I	0.50 [−0.24–1.24; *P* = 0.03; *k* = 2]	1.37 [0.97–1.77; *P* < 0.01; *k* = 39]	0.00 [−0.36–0.36; N/A; *k* = 1]
** **SMNΔ7	1.62 [1.36–1.88; *P* < 0.01; *k* = 53]	0.75 [0.59–0.90; *P* < 0.01; *k* = 96]	1.06 [0.85–1.28; *P* < 0.01; *k* = 58]
** **Taiwanese	N/A [*k* = 0]	1.51 [1.29–1.72; *P* < 0.01; *k* = 89]	1.07 [0.68–1.46; *P* < 0.01; *k* = 9]
** **Smn2B	1.12 [0.73–1.51; *P* < 0.01; *k* = 10]	N/A [*k* = 0]	0.84 [0.36–1.32; *P* < 0.01; *k* = 9]
** **Burgheron	N/A [*k* = 0]	0.33 [0.14–0.52; *P* < 0.01; *k* = 2]	N/A [*k* = 0]
** **Other	N/A [*k* = 0]	0.26 [−0.69–1.20; *P* < 0.01; *k* = 4]	N/A [*k* = 0]
Route of administration			
Subgroup differences?	*P* < 0.01	*P* < 0.01	*P* < 0.01
** **CNS	1.73 [1.42 −2.04; *P* < 0.01; *k* = 33]	0.62 [0.45–0.78; *P* < 0.01; *k* = 103]	0.16 [−0.15–0.47; *P* = 0.21; *k* = 2]
** **Systemic	1.13 [0.83–1.42; *P* < 0.01; *k* = 29]	1.53 [1.34–1.72; *P* < 0.01; *k* = 116]	1.05 [0.87–1.22; *P* < 0.01; *k* = 75]
** **Both	2.83 [2.55–3.12; *P* = 0.47; *k* = 3]	1.70 [1.32–2.08; *P* < 0.01; *k* = 11]	N/A [*k* = 0]

*K*, number of comparisons per group. Values indicate effect size [95% CI].

We also explored additional factors as potential sources of heterogeneity, such as dose, frequency of administration, sex of animal and transgenic model used. Upon inspection of the dataset, dosage of SMN replacement therapies varied wildly and could thus not be reliably compared between different therapy types. For example, viral vector delivery ranged from 1.7 × 10^10^ to 8 × 10^11^ vector genomes; ASOs were delivered at doses between 10 and 400 µg/g and small molecule therapy was given between 0.03 and 30 µg/g. The sex of animals used in these studies was rarely described. Because of these constraints, these two parameters were not examined as sources of heterogeneity, in a deviation from our protocol. Additionally, route of administration (CNS delivery versus systemic delivery) was considered an informative comparison to add to subgroup analyses as a *post hoc* deviation from protocol.

Frequency of administration was only found to be a significant source of heterogeneity in the ASO treatment group, where multiple doses showed a stronger effect on survival (1.48 [1.28–1.68], *P* < 0.01), although small molecule treatment was always given across multiple doses. The route of administration was a significant source of heterogeneity across all three treatment groups, with systemic treatment showing a stronger effect on survival in both ASO and small molecule treatment, while viral vectors had better outcomes following CNS delivery (Table 1). Furthermore, we found that treatment regimes delivering ASOs both systemically and directly to the CNS were most effective in terms of survival (1.70 [1.32–2.08], *P* < 0.01). Choice of SMA model used was a significant source of heterogeneity across all three treatment groups. While this may not be as useful when translating to a clinical context, these results are important to consider when interpreting preclinical studies. Within the viral vector treatment group, the strongest effect on survival was found in the SMNΔ7 SMA model, followed by the Smn2B/- model, whereas the SMNΔ7 model was less responsive to ASO therapy than the Taiwanese mouse model. Within the small molecule treatment group, both the SMNΔ7 and Taiwanese mouse models showed similar responses with the Smn2B/- model showing a weaker effect on survival (Table 1).

**Figure 4:**
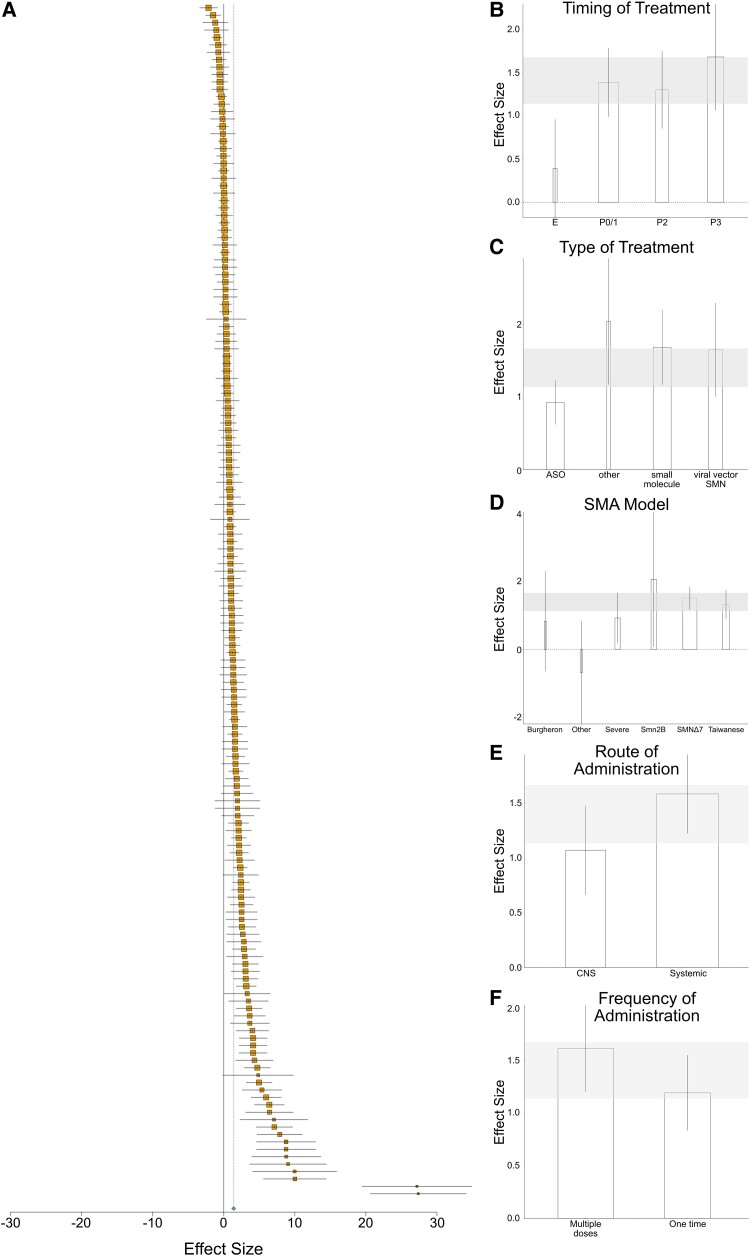
**Effect of SMN replacement therapies on body weight**. (**A**) Forest plot showing all comparisons (*k* = 161) of treated versus untreated SMA mice on the day of maximum body weight of the untreated group, with an effect favouring intervention (blue diamond—1.40 [1.14–1.67], *P* < 0.001). The *x*-axis shows effect size, bars represent 95% confidence intervals, size of yellow box represents number of animals in the study. (**B–F**) Histograms of subgroup analyses. Width of bars indicate number of comparisons per group. Grey shading indicates overall effect of SMN replacement therapies on body weight. (B) Subgroup analysis showing that timing of treatment was not a significant source of heterogeneity (*P* = 0.011). Embryonic treatment (E; 0.39 [−0.18–0.96], *k* = 3); post-natal day (*P*) 0/1 (1.38 [0.99–1.78], *k* = 79); post-natal day (*P*) 2 (1.30 [0.85–1.74], *k* = 31); post-natal day 3 or later (P3+) (1.68 [1.06–2.29], *k* = 48). (C) Subgroup analysis showing that type of treatment was a significant source of heterogeneity on body weight (*P* = 0.0079). ASO (0.66 [0.50−0.82], *k* = 57); other (2.04 [1.18–2.91], *k* = 6); small molecules (0.88 [0.71–1.05], *k* = 57); viral vectors (1.65 [1.01–2.30], *k* = 41). (D) Subgroup analysis showing SMA model was not a significant source of heterogeneity on body weight (*P* = 0.0775). Burgheron intermediate model (0.84 [−0.64–2.31], *k* = 2); other (−0.67 [−2.19–0.84], *k* = 2); severe (0.93 [0.19–1.68], *k* = 10); Smn2B/− (2.07 [0.083–4.06], *k* = 14); SMNΔ7 (1.52 [1.18–1.86], *k* = 99); Taiwanese (1.33 [0.91–1.76], *k* = 34). (E) Subgroup analysis showing route of administration was not a significant source of heterogeneity on body weight (*P* = 0.0635). Systemic (0.90 [0.77–1.02], *k* = 114); CNS-directed (0.59 [0.41–0.76], *k* = 47). (F) Subgroup analysis showing frequency of administration was not a significant source of heterogeneity on body weight (*P* = 0.130). Multiple doses (1.61 [1.20–2.02], *k* = 93); one time dose (1.19 [0.83–1.55], *k* = 68).

As a secondary outcome measure, we assessed the impact of SMN replacement therapies on body weight. Increase in body weight is a standard phenotype in both preclinical and clinical studies to assess efficacy of SMN replacement therapies. Body weight data extracted from each study was taken on the day of maximum body weight for untreated SMA control groups. 161 body weight comparisons were included for meta-analysis (Fig. 4A). These results showed that treatment with SMN replacement therapies had a positive effect on body weight compared to control, to a factor of 1.40 (95% CI 1.14–1.67]; *P* < 0.001). There was significant heterogeneity within this dataset (*I*^2^ = 78%). Subgroup analysis investigated timing of treatment, type of treatment, SMA model, route of administration and frequency of administration as possible sources of heterogeneity (Fig. 4B–F).

When data were stratified according to subgroup, timing of SMN treatment was not found to be a significant source of heterogeneity (*P* = 0.011). However, type of treatment was a significant source of heterogeneity (*P* = 0.0079). The largest effect on body weight was seen with the ‘other’ treatment group (2.04 [1.18–2.91]) albeit with a small number of comparisons (*k* = 6). Viral vectors and small molecule treatments showed similar effects on body weight (1.65 [1.01–2.30] and 1.69 [1.17–2.20], respectively), while ASO treatment had the smallest effect on body weight (0.93 [0.62–1.23]). Route of administration, SMA model and frequency of administration were not found to be sources of heterogeneity (Fig. 4).

**Figure 5:**
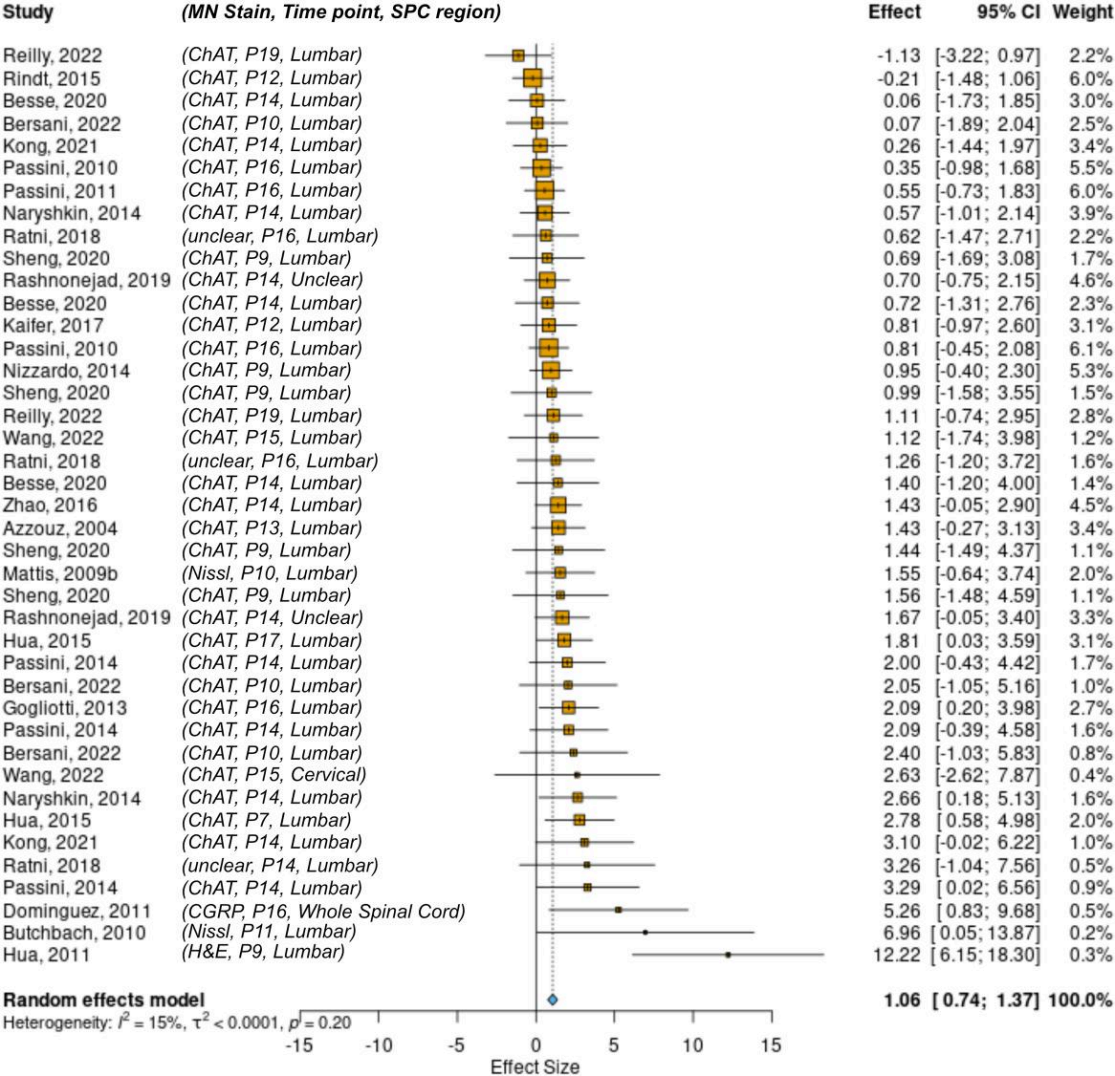
**The effect of SMN replacement therapies on motor neuron counts**. Forest plot showing that SMN replacement therapies show a significant effect favouring intervention on motor neuron count 1.06 (0.74–1.37; *P* < 0.001; *k* = 41). MN Stain, type of stain used to identify motor neurons; Time point, postnatal day of dissection; SPC region, target region of spinal cord for MN analysis; ChAT, choline acetyltransferase; CGRP, calcitonin gene-related peptide; H&E, haematoxylin and eosin.

Another secondary outcome measure examined was motor neuron number in the spinal cord. A total of 41 comparisons were included and analysed. A forest plot of the results is shown in Fig. 5, with SMN replacement therapies improving motor neuron counts by a factor of 1.06 (0.74–1.37; *P* < 0.001). While there was low heterogeneity in this dataset (*I*^2^ = 15%), there was experimental variability between studies in terms of method of motor neuron identification, time point of analysis and region of spinal cord investigated, although this was predominantly the lumbar region. This variability is highlighted in the second column of Fig. 5. Whilst most of this heterogeneity will be accounted for by the comparison to the untreated control within the study, these variables should be taken into account when interpreting the result. There were too few comparisons to split the dataset according to sources of heterogeneity.

**Figure 6:**
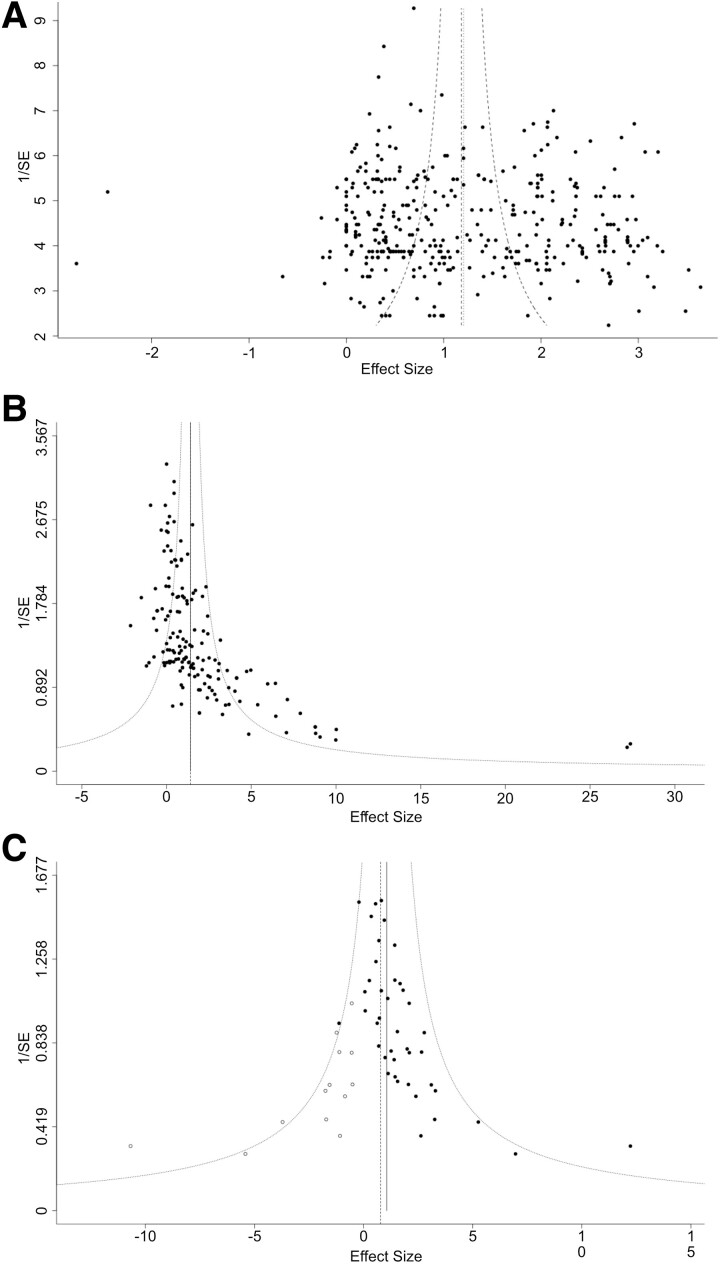
**Funnel plots with trim and fill analysis to assess for publication bias**. (**A**) Funnel plot of effect size versus 1/SE (standard error) for survival analysis shows a symmetrical plot (Egger’s test, *P* = 0.30). (**B**) Funnel plot of effect size versus 1/SE (standard error) for body weight analysis shows an asymmetrical plot (Egger’s test, *P* < 0.001) with Trim and Fill analysis estimating 0 missed studies. (**C**) Funnel plot of effect size versus 1/SE (standard error) for motor neuron count analysis shows an asymmetrical plot (Egger’s test, *P* = 0.011) with Trim and Fill analysis estimating 13 missed studies (white points).

Finally, funnel plots of effect size versus inverse standard error were used to assess publication bias (Fig. 6). The primary outcome of survival shows a symmetrical funnel plot (Fig. 6A) with no significant evidence of publication bias (Egger’s test, *P* = 0.30). Meanwhile, the secondary outcome of changes in body weight shows a strongly asymmetrical funnel plot (Fig. 6B, Egger’s test *P* < 0.001) although trim and fill analysis did not identify missing studies. Motor neuron counts also show an asymmetrical funnel plot, with a trim and fill analysis estimating 13 missing studies on the left side (Fig. 6C, Egger’s test *P* = 0.011).

## Discussion

In the era of gene therapies, ensuring that children born with severe SMA receive the best possible care means advocating for the right treatment at the right time. Solid evidence needs to be gathered from available data to determine the best therapeutic time window. Approved SMN-dependent therapies are all still in their infancy and clinical data comparing timings of treatment administration remain sparse. To date, the results of the SPR1NT (for Zolgensma) and interim results of the NURTURE (for Spinraza) clinical trials are the only data available from human patients.^11-13^ They both indicate higher therapeutic benefits with earlier, pre-symptomatic treatment. Here, we explored the wealth of data available from preclinical research on these therapies, to assess timing of treatment administration and inform future research and therapeutic strategies.

Following established methodologies and gold standard preclinical systematic review guidelines, we performed a meta-analysis on 78 studies, with 386 comparisons reporting results of SMN-dependent treatments in mouse models of SMA.^17,19^ Overall, effect sizes for median survival following treatment favoured intervention for all treatment types (viral vectors, ASOs and small molecules). Timing of treatment was a source of heterogeneity, with the biggest benefit observed for prenatal intervention, followed by treatment at the day of birth. This supports the claim that earlier treatment results in better survival outcomes. However, embryonic data were limited, with only three or four comparisons available for each treatment type, thus limiting the power of the meta-analysis for this subgroup. Viral vectors showed the biggest effect size for such prenatal treatment, whereas ASO comparisons reported in a single study had extremely small effect sizes. Injection of SMN therapies directly into mouse embryos is technically challenging and few studies exist that focus on this approach. SMN-boosting small molecules such as risdiplam were developed more recently than ASOs and viral vectors. As such, we found fewer datapoints available to interpret, especially at early developmental stages. This may in part explain the fact that timing of intervention was not a significant source of heterogeneity for such compounds.

For our secondary outcomes, we analysed body weights on the day of maximum reported value for untreated SMA pups and motor neuron counts. Not all studies reported these outcomes, particularly motor neuron counts, meaning that we could not perform subgroup analyses. Within the body weight meta-analysis, only the type of treatment was shown to be a significant source of heterogeneity, with viral vector delivery and small molecule therapy having a greater effect than ASO treatment. Interestingly, the effect of SMN replacement therapies on body weight was the same across time points of postnatal delivery.

The main limitation of this review came from the lack of reporting of risk of bias factors, which limits what we can infer from the results. This is a common issue in preclinical research, associated with over-estimation of effect sizes and lack of replicability,^23^ which updated reporting guidelines are aiming to curb.^24,25^ Future studies will likely report factors such as sex, blinding, randomization, confounding factors and housing conditions on a more regular basis, allowing for more refined conclusions to be drawn. By highlighting these issues, we are hoping this systematic review will drive an increase in the awareness of risk of bias factors and their reporting, thereby increasing the validity of preclinical SMA studies.

When considering our primary outcome of survival, this study shows that early treatment of current SMN-dependent therapies has the greatest effect on median survival in systemic mouse models of SMA. This should serve to inform future preclinical studies testing therapies for SMA, as only early administration yields long lifespan extension. Additionally, our study provides valuable insight into the state of SMA preclinical research, showing that it corresponds to the trends seen in clinical settings and thus confirming the value and importance of SMA mouse models. With this additional evidence from almost 20 years of SMA research, a strong argument can be made to expand the systematic use of NBS to detect SMA at birth. Timing of treatment is crucial and early access can only be delivered through widely adopted NBS programmes, as already implemented by some countries such as Belgium, Australia, Canada, Italy and Japan.^16,26^

## Supplementary Material

fcae267_Supplementary_Data

## Data Availability

The protocol detailing the methods was pre-registered on PROSPERO www.crd.york.ac.uk/prospero/display_record.php? ID=CRD42020200180. The complete dataset and the R scripts are available on the University of Edinburgh’s DataShare repository https://datashare.ed.ac.uk/handle/10283/8546 (https://doi.org/10.7488/ds/7526).
