# Supplementary material for: Timing of SMN replacement therapies in mouse models of spinal muscular atrophy: a systematic review and meta-analysis
Source: Brain Commun. 2024 Aug 12;6(4):fcae267. doi: 10.1093/braincomms/fcae267 (PMC11342241; doi:10.1093/braincomms/fcae267)
Supplement: fcae267_Supplementary_Data [file fcae267_supplementary_data.pdf]

Supplementary Material

Search terms:
We used the following search strategy for PubMed Search - (Spinal muscular atrophy [MeSH] OR Spinal muscular atrophy [Aab]) AND (animals[Aab] OR animal[Aab] OR mice[Tiab] OR mus[Tiab] OR mouse[Tiab] OR murine[Tiab] OR murinae[Tiab] OR muridae[Tiab] OR rodenAa[Tiab] OR rodent[Tiab] OR rodents[Tiab] OR mouse [MeSH] OR murine [MeSH] OR mice [MeSH]);
Web of Science - “Spinal muscular atrophy” AND (mouse OR mice OR murine OR animals OR animal OR mus OR murinae OR muridae OR rodenAa OR rodent OR rodents);
EMBASE - Spinal muscular atrophy AND (mouse OR mice OR murine OR animals OR animal OR mus OR murinae OR muridae OR rodenAa OR rodent OR rodents); Scopus - ("Spinal muscular atrophy" AND (mouse OR mice OR murine OR animals OR animal OR mus OR murinae)).

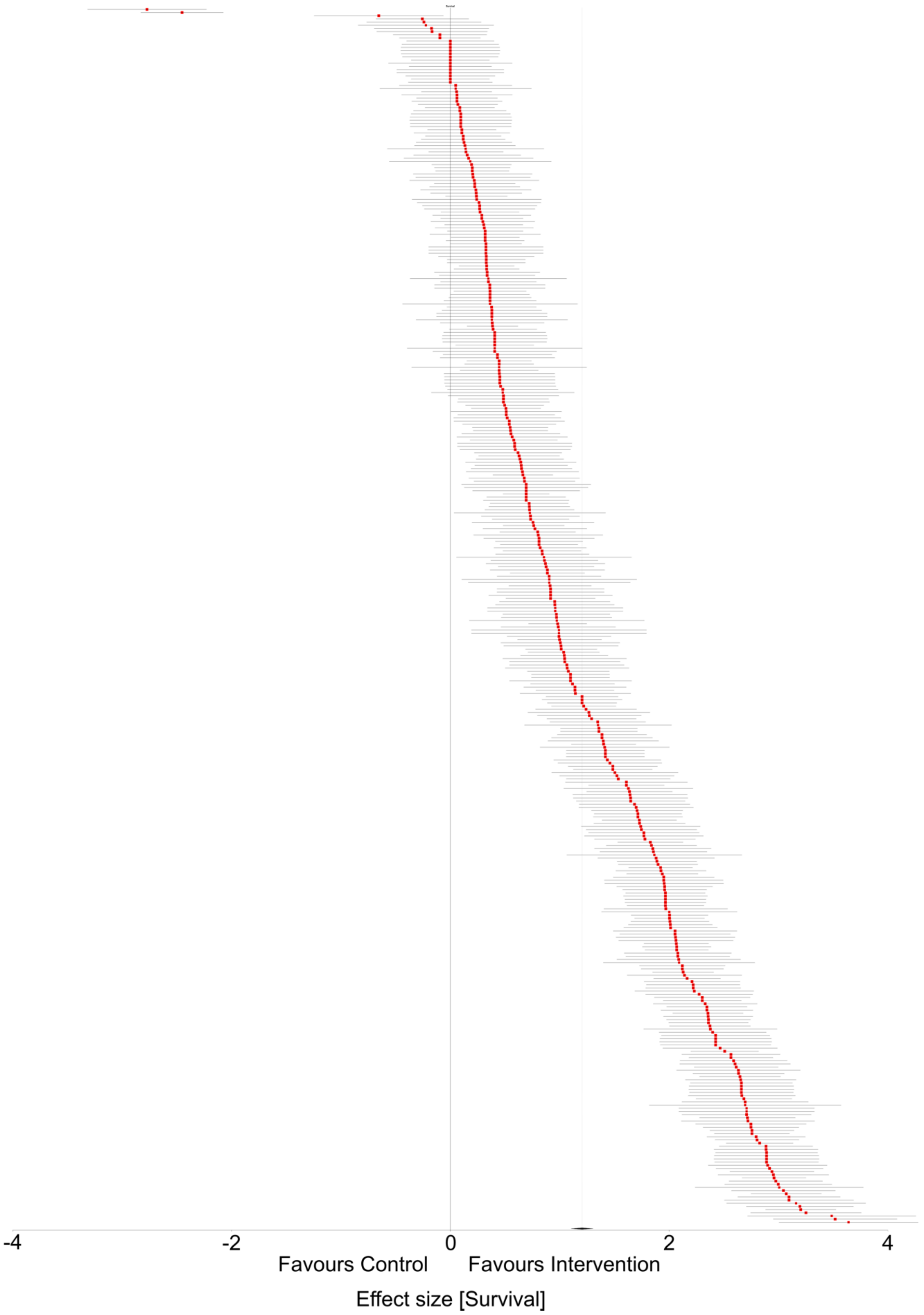

Supplementary Figure 1. Forest plot of survival following SMN replacement therapy in all included comparisons, order by effect sizes.

Supplementary Table 1. Summary of the studies included in the meta-analysis

TITLE	AUTHOR	YEAR	SMA MODEL	TYPE OF THERAPY	MIN. NO. OF ANIMALS PER GROUP (SURVIVAL)	FIRST DAY OF TREATMENT
LENTIVECTOR-MEDIATED SMN REPLACEMENT IN A MOUSE MODEL OF SPINAL MUSCULAR ATROPHY [1]	Azzouz	2004	SMNΔ7	Viral vector (Lenti-SMN)	5	P2
DELIVERY OF BIFUNCTIONAL RNAS THAT TARGET AN INTRONIC REPRESSOR AND INCREASE SMN LEVELS IN AN ANIMAL MODEL OF SPINAL MUSCULAR ATROPHY [2]	Baughan	2009	Burghes' severe	Antisense oligonucleotide (bifunctional RNAs)	11	P2
DELIVERY OF A READ-THROUGH INDUCING COMPOUND TC007 LESSENS THE SEVERITY OF A SPINAL MUSCULAR ATROPHY ANIMAL MODEL [3]	Mattis	2009a	SMNΔ7	Small molecule (TC007)	16	P3
SUBCUTANEOUS ADMINISTRATION OF TC007 REDUCES DISEASE SEVERITY IN AN ANIMAL MODEL OF SMA[4]	Mattis	2009b	SMNΔ7	Small molecule (TC007)	8	P2
EFFECTS OF 24-DIAMINOQUINAZOLINE DERIVATIVES ON SMN EXPRESSION AND PHENOTYPE IN A MOUSE MODEL FOR SPINAL MUSCULAR ATROPHY [5]	Butchbach	2010	SMNΔ7	Small molecule (D156844)	14	E11.5; P4
TRANS-SPLICING-MEDIATED IMPROVEMENT IN A SEVERE MOUSE MODEL OF SPINAL MUSCULAR ATROPHY [6]	Coady	2010	Burghes' severe	Trans-splicing plasmid	15	P2
RESCUE OF THE SPINAL MUSCULAR ATROPHY PHENOTYPE IN A MOUSE MODEL BY EARLY POSTNATAL DELIVERY OF SMN [7]	Foust	2010	SMNΔ7	Viral vector (AAV9-SMN)	9	P1; P2; P5; P10
CNS-TARGETED GENE THERAPY IMPROVES SURVIVAL AND MOTOR FUNCTION IN A MOUSE MODEL OF SPINAL MUSCULAR ATROPHY [8]	Passini	2010	SMNΔ7	Viral vector (AAV8-SMN)	10	P0
SYSTEMIC DELIVERY OF SCAAV9 EXPRESSING SMN PROLONGS SURVIVAL IN A MODEL OF SPINAL MUSCULAR ATROPHY [9]	Valori	2010	SMNΔ7	Viral vector (AAV9-SMN)	9	P1
INTRAVENOUS SCAAV9 DELIVERY OF A CODON-OPTIMIZED SMN1 SEQUENCE RESCUES SMA MICE [10]	Dominguez	2011	SMNΔ7	Viral vector (AAV9-SMN)	3	P1
PERIPHERAL SMN RESTORATION IS ESSENTIAL FOR LONG-TERM RESCUE OF A SEVERE SPINAL MUSCULAR ATROPHY MOUSE MODEL [11]	Hua	2011	Burghes' severe	Antisense oligonucleotide (2'-O-Me)	12	E15; P1
ANTISENSE OLIGONUCLEOTIDES DELIVERED TO THE MOUSE CNS AMELIORATE SYMPTOMS OF SEVERE SPINAL MUSCULAR ATROPHY [12]	Passini	2011	SMNΔ7	Antisense oligonucleotide (2'-O-Me)	5	P0
COMBINATION OF SMN TRANS-SPLICING AND A NEUROTROPHIC FACTOR INCREASES THE LIFE SPAN AND BODY MASS IN A SEVERE MODEL OF SPINAL MUSCULAR ATROPHY [13]	Shababi	2011	Burghes' severe	Trans-splicing plasmid	10	P2
DIRECT CENTRAL NERVOUS SYSTEM DELIVERY PROVIDES ENHANCED PROTECTION FOLLOWING VECTOR MEDIATED GENE REPLACEMENT IN A SEVERE MODEL OF SPINAL MUSCULAR ATROPHY [14]	Glascock	2012a	SMNΔ7	Viral vector (scAAV9-SMN)	4	P1
DECREASING DISEASE SEVERITY IN SYMPTOMATIC SMN(-/-); SMN2(+/+) SPINAL MUSCULAR ATROPHY MICE FOLLOWING SCAAV9-SMN DELIVERY [15]	Glascock	2012b	Burghes' severe	Viral vector (scAAV9-SMN)	6	P1

ANALYSIS OF A READ-THROUGH PROMOTING COMPOUND IN A SEVERE MOUSE MODEL OF SPINAL MUSCULAR ATROPHY [16]	Mattis	2012	Burghes' severe	Small molecule (TC007)	13	P1
BIFUNCTIONAL RNAS TARGETING THE INTRONIC SPLICING SILENCER N1 INCREASE SMN LEVELS AND REDUCE DISEASE SEVERITY IN AN ANIMAL MODEL OF SPINAL MUSCULAR ATROPHY [17]	Osman	2012	SMNΔ7	Antisense oligonucleotide (2'-O-Me)	10	P1
A SINGLE ADMINISTRATION OF MORPHOLINO ANTISENSE OLIGOMER RESCUES SPINAL MUSCULAR ATROPHY IN MOUSE [18]	Porensky	2012	SMNΔ7	Antisense oligonucleotide (PMO)	3	P0; P4
PARTIAL RESTORATION OF CARDIO-VASCULAR DEFECTS IN A RESCUED SEVERE MODEL OF SPINAL MUSCULAR ATROPHY [19]	Shababi	2012	SMNΔ7	Viral vector (scAAV9-SMN)	10	P2
INTRAMUSCULAR SCAAV9-SMN INJECTION MEDIATES WIDESPREAD GENE DELIVERY TO THE SPINAL CORD AND DECREASES DISEASE SEVERITY IN SMA MICE [20]	Benkhelifa-Ziyyat	2013	SMNΔ7	Viral vector (scAAV9-SMN)	5	P0
ENHANCEMENT OF SMN PROTEIN LEVELS IN A MOUSE MODEL OF SPINAL MUSCULAR ATROPHY USING NOVEL DRUG-LIKE COMPOUNDS [21]	Cherry	2013	SMNΔ7	Small molecule (LDN-76070)	10	P2
THE DCPS INHIBITOR RG3039 IMPROVES SURVIVAL FUNCTION AND MOTOR UNIT PATHOLOGIES IN TWO SMA MOUSE MODELS [22]	Gogliotti	2013	Smn2B/- Taiwanese	Small molecule (RG3039)	3	P4; P11
IMPROVED ANTISENSE OLIGONUCLEOTIDE DESIGN TO SUPPRESS ABERRANT SMN2 GENE TRANSCRIPT PROCESSING: TOWARDS A TREATMENT FOR SPINAL MUSCULAR ATROPHY [23]	Mitrpant	2013	SMNΔ7	Antisense oligonucleotide (PMO)	4	P0
A NOVEL MORPHOLINO OLIGOMER TARGETING ISS-N1 IMPROVES RESCUE OF SEVERE SPINAL MUSCULAR ATROPHY TRANSGENIC MICE [24]	Zhou	2013	Taiwanese	Antisense oligonucleotide (PMO)	5	P0
THE EFFECT OF DIET ON THE PROTECTIVE ACTION OF D156844 OBSERVED IN SPINAL MUSCULAR ATROPHY MICE [25]	Butchbach	2014	SMNΔ7	Small molecule (D156844)	12	P4
A SHORT ANTISENSE OLIGONUCLEOTIDE AMELIORATES SYMPTOMS OF SEVERE MOUSE MODELS OF SPINAL MUSCULAR ATROPHY [26]	Keil	2014	Taiwanese 5058-Hemi hybrid	Antisense oligonucleotide (3UP8i)	5	P0
SMN2 SPLICING MODIFIERS IMPROVE MOTOR FUNCTION AND LONGEVITY IN MICE WITH SPINAL MUSCULAR ATROPHY [27]	Naryshkin	2014	SMNΔ7	Small molecule (SMN-C3; SMN-C2)	15	P3; P4
EFFECT OF COMBINED SYSTEMIC AND LOCAL MORPHOLINO TREATMENT ON THE SPINAL MUSCULAR ATROPHY DELTA7 MOUSE MODEL PHENOTYPE [28]	Nizzardo	2014	SMNΔ7	Antisense oligonucleotide (PMO)	3	P0; P5
MORPHOLINO ANTISENSE OLIGONUCLEOTIDES TARGETING INTRONIC REPRESSOR ELEMENT1 IMPROVE PHENOTYPE IN SMA MOUSE MODELS [29]	Osman	2014	Taiwanese SMN^{RT}	Antisense oligonucleotide (PMO)	5	P2
TRANSLATIONAL FIDELITY OF INTRATHECAL DELIVERY OF SELF-COMPLEMENTARY AAV9-SURVIVAL MOTOR NEURON 1 FOR SPINAL MUSCULAR ATROPHY [30]	Passini	2014	SMNΔ7	Viral vector (scAAV9-SMN)	4	P0
DEFINING THE THERAPEUTIC WINDOW IN A SEVERE ANIMAL MODEL OF SPINAL MUSCULAR ATROPHY [31]	Robbins	2014	SMNΔ7	Viral vector (scAAV9-SMN)	8	P2; P3; P4; P8

SYSTEMIC POSTSYMPTOMATIC ANTISENSE OLIGONUCLEOTIDE RESCUES MOTOR UNIT MATURATION DELAY IN A NEW MOUSE MODEL FOR TYPE II/III SPINAL MUSCULAR ATROPHY [32]	Bogdanik	2015	Burgheron intermediate	Antisense oligonucleotide (2'-O-Me)	6	P10; P25
MOTOR NEURON CELL-NONAUTONOMOUS RESCUE OF SPINAL MUSCULAR ATROPHY PHENOTYPES IN MILD AND SEVERE TRANSGENIC MOUSE MODELS [33]	Hua	2015	Taiwanese	Antisense oligonucleotide (2'-O-Me)	6	P0
IMPROVING SINGLE INJECTION CSF DELIVERY OF AAV9-MEDIATED GENE THERAPY FOR SMA: A DOSE-RESPONSE STUDY IN MICE AND NONHUMAN PRIMATES [34]	Meyer	2015	SMNΔ7	Viral vector (AAV9-SMN)	10	P1
SMN2 SPLICE MODULATORS ENHANCE U1-PRE-MRNA ASSOCIATION AND RESCUE SMA MICE [35]	Palacino	2015	SMNΔ7	Small molecule (NVS-SM1)	8	P3
ASTROCYTES INFLUENCE THE SEVERITY OF SPINAL MUSCULAR ATROPHY [36]	Rindt	2015	SMNΔ7 Smn2B/-	Viral vector (AAV9-SMN)	4	P1
REPEATED LOW DOSES OF MORPHOLINO ANTISENSE OLIGOMER: AN INTERMEDIATE MOUSE MODEL OF SPINAL MUSCULAR ATROPHY TO EXPLORE THE WINDOW OF THERAPEUTIC RESPONSE [37]	Zhou	2015	Taiwanese	Antisense oligonucleotide (PMO)	3	P0
EFFICACY AND BIODISTRIBUTION ANALYSIS OF INTRACEREBROVENTRICULAR ADMINISTRATION OF AN OPTIMIZED SCAAV9-SMN1 VECTOR IN A MOUSE MODEL OF SPINAL MUSCULAR ATROPHY [38]	Armbruster	2016	SMNΔ7	Viral vector (AAV9-SMN)	8	P0
THE NEUROMUSCULAR IMPACT OF SYMPTOMATIC SMN RESTORATION IN A MOUSE MODEL OF SPINAL MUSCULAR ATROPHY [39]	Arnold	2016	SMNΔ7	Antisense oligonucleotide (PMO)	5	P4; P6
PHARMACOLOGICALLY INDUCED MOUSE MODEL OF ADULT SPINAL MUSCULAR ATROPHY TO EVALUATE EFFECTIVENESS OF THERAPEUTICS AFTER DISEASE ONSET [40]	Feng	2016	SMNΔ7	Small molecule (SMN-C3)	23	P3
SYSTEMIC PEPTIDE-MEDIATED OLIGONUCLEOTIDE THERAPY IMPROVES LONG-TERM SURVIVAL IN SPINAL MUSCULAR ATROPHY [41]	Hammond	2016	Taiwanese	Antisense oligonucleotide (PMO)	3	P0
THE POWER OF HUMAN PROTECTIVE MODIFIERS: PLS3 AND CORO1C UNRAVEL IMPAIRED ENDOCYTOSIS IN SPINAL MUSCULAR ATROPHY AND RESCUE SMA PHENOTYPE [42]	Hosseinibarkooie	2016	Taiwanese	Antisense oligonucleotide (2'-O-Me)	8	P2
SELECTIVE NEUROMUSCULAR DENERVATION IN TAIWANESE SEVERE SMA MOUSE CAN BE REVERSED BY MORPHOLINO ANTISENSE OLIGONUCLEOTIDES [43]	Lin	2016	Taiwanese	Antisense oligonucleotide (PMO)	10	P0
SOMATIC THERAPY OF A MOUSE SMA MODEL WITH A U7 SNRNA GENE CORRECTING SMN2 SPLICING [44]	Odermatt	2016	SMNΔ7	Viral vector (scAAV9-U7; scAAV9-SMN)	3	P0
OPTIMIZATION OF MORPHOLINO ANTISENSE OLIGONUCLEOTIDES TARGETING THE INTRONIC REPRESSOR ELEMENT1 IN SPINAL MUSCULAR ATROPHY [45]	Osman	2016	SMNΔ7	Antisense oligonucleotide (PMO)	4	P1
SPECIFIC CORRECTION OF ALTERNATIVE SURVIVAL MOTOR NEURON 2 SPLICING BY SMALL MOLECULES: DISCOVERY OF A POTENTIAL NOVEL MEDICINE TO TREAT SPINAL MUSCULAR ATROPHY [46]	Ratni	2016	SMNΔ7	Small molecule (Compound 3, 4 & 5)	15	P3

DISCOVERY AND OPTIMIZATION OF SMALL MOLECULE SPLICING MODIFIERS OF SURVIVAL MOTOR NEURON 2 AS A TREATMENT FOR SPINAL MUSCULAR ATROPHY [47]	Woll	2016	SMNΔ7	Small molecule (Compound 9, 14 & 20)	11	P3
PHARMACOKINETICS PHARMACODYNAMICS AND EFFICACY OF A SMALL-MOLECULE SMN2 SPLICING MODIFIER IN MOUSE MODELS OF SPINAL MUSCULAR ATROPHY [48]	Zhao	2016	SMNΔ7	Small molecule (SMN-C1)	15	P3
THE ANTISENSE TRANSCRIPT SMN-AS1 REGULATES SMN EXPRESSION AND IS A NOVEL THERAPEUTIC TARGET FOR SPINAL MUSCULAR ATROPHY [49]	d'Ydewalle	2017	SMNΔ7	Antisense oligonucleotide (2'-O-Me)	15	P1
PLASTIN-3 EXTENDS SURVIVAL AND REDUCES SEVERITY IN MOUSE MODELS OF SPINAL MUSCULAR ATROPHY [50]	Kaifer	2017	SMNΔ7	Antisense oligonucleotide (PMO)	5	P1
ANALYSIS OF AZITHROMYCIN MONOHYDRATE AS A SINGLE OR A COMBINATORIAL THERAPY IN A MOUSE MODEL OF SEVERE SPINAL MUSCULAR ATROPHY [51]	Osman	2017	SMNΔ7	Antisense oligonucleotide (PMO)	6	P1
IDENTIFICATION OF A PEPTIDE FOR SYSTEMIC BRAIN DELIVERY OF A MORPHOLINO OLIGONUCLEOTIDE IN MOUSE MODELS OF SPINAL MUSCULAR ATROPHY [52]	Shabanpoor	2017	Taiwanese	Antisense oligonucleotide (PMO)	8	P0
SELF-OLIGOMERIZATION REGULATES STABILITY OF SURVIVAL MOTOR NEURON PROTEIN ISOFORMS BY SEQUESTERING AN SCF(SLMB) DEGRON [53]	Gray	2018	Smn2B/-	Viral vector (AAV9-SMN)	5	P1
CHP1 REDUCTION AMELIORATES SPINAL MUSCULAR ATROPHY PATHOLOGY BY RESTORING CALCINEURIN ACTIVITY AND ENDOCYTOSIS [54]	Janzen	2018	Taiwanese	Antisense oligonucleotide (2'-O-Me)	19	P2
DISCOVERY OF RISDIPLAM A SELECTIVE SURVIVAL OF MOTOR NEURON-2 (SMN2) GENE SPLICING MODIFIER FOR THE TREATMENT OF SPINAL MUSCULAR ATROPHY (SMA) [55]	Ratni	2018	SMNΔ7	Small molecule (RG7916)	10	P3
RESCUE OF SPINAL MUSCULAR ATROPHY MOUSE MODELS WITH AAV9-EXON-SPECIFIC U1 SNRNA [56]	Donadon	2019	Taiwanese	Viral vector (AAV9-ExspeU1)	3	P0
FUNCTIONAL CHARACTERIZATION OF SMN EVOLUTION IN MOUSE MODELS OF SMA [57]	Osman	2019a	SMNΔ7	Viral vector (AAV9-SMN)	6	P2
INTRAPERITONEAL DELIVERY OF A NOVEL DRUG-LIKE COMPOUND IMPROVES DISEASE SEVERITY IN SEVERE AND INTERMEDIATE MOUSE MODELS OF SPINAL MUSCULAR ATROPHY [58]	Osman	2019b	SMNΔ7<br>Smn2B/-	Small molecule (LDN-76;LDN-2014)	6	P2
FETAL GENE THERAPY USING A SINGLE INJECTION OF RECOMBINANT AAV9 RESCUED SMA PHENOTYPE IN MICE [59]	Rashnonejad	2019	SMNΔ7	Viral vector (scAAV9-SMN; ssAAV9-SMN)	5	E15
AAV9-MEDIATED EXPRESSION OF SMN RESTRICTED TO NEURONS DOES NOT RESCUE THE SPINAL MUSCULAR ATROPHY PHENOTYPE IN MICE [60]	Besse	2020	SMNΔ7	Viral vector (AAV9-SMN)	3	P1
DISCOVERY OF A CNS PENETRANT SMALL MOLECULE SMN2 SPLICING MODULATOR WITH IMPROVED TOLERABILITY FOR SPINAL MUSCULAR ATROPHY [61]	Ando	2020	SMNΔ7	Small molecule (TEC-1)	3	P2
COMPARISON OF THE EFFICACY OF MOE AND PMO MODIFICATIONS OF SYSTEMIC ANTISENSE OLIGONUCLEOTIDES IN A SEVERE SMA MOUSE MODEL [62]	Sheng	2020	Taiwanese	Antisense oligonucleotide (2'-O-Me; PMO)	14	P0

MYOSTATIN INHIBITION IN COMBINATION WITH ANTISENSE OLIGONUCLEOTIDE THERAPY IMPROVES OUTCOMES IN SPINAL MUSCULAR ATROPHY [63]	Zhou	2020	Taiwanese	Antisense oligonucleotide (PMO)	7	P0
SHORT-DURATION SPLICE PROMOTING COMPOUND ENABLES A TUNABLE MOUSE MODEL OF SPINAL MUSCULAR ATROPHY [64]	Rietz	2021	Taiwanese SMNΔ7	Small molecule (NVS-SM2)	4	P2; P6
IMPAIRED PRENATAL MOTOR AXON DEVELOPMENT NECESSITATES EARLY THERAPEUTIC INTERVENTION IN SEVERE SMA [65]	Kong	2021	SMNΔ7	Small molecule (SMN-C3)	3	E9.5; E13.5; P1
GAIN OF TOXIC FUNCTION BY LONG-TERM AAV9-MEDIATED SMN OVEREXPRESSION IN THE SENSORIMOTOR CIRCUIT [66]	Van Alstyne	2021	SMNΔ7	Viral vector (AAV9-SMN)	16	P0
MESYL PHOSPHORAMIDATE OLIGONUCLEOTIDES AS POTENTIAL SPLICE-SWITCHING AGENTS: IMPACT OF BACKBONE STRUCTURE ON ACTIVITY AND INTRACELLULAR LOCALIZATION [67]	Hammond	2021	Taiwanese	Antisense oligonucleotide (Nus-MOE-mes/thio)	2	P0
DUAL SMN INDUCING THERAPIES CAN RESCUE SURVIVAL AND MOTOR UNIT FUNCTION IN SYMPTOMATIC Δ7SMA MICE[68]	Kray	2021	SMNΔ7	Antisense oligonucleotide (PMO) ± small molecule (RG7800)	12	P2; P4; P6
SMN PROTEIN IS REQUIRED THROUGHOUT LIFE TO PREVENT SPINAL MUSCULAR ATROPHY DISEASE PROGRESSION[69]	Zhao	2021	SMNΔ7	Small molecule (PTC-SSH)	16	P3
CELL-PENETRATING PEPTIDE-CONJUGATED MORPHOLINO RESCUES SMA IN A SYMPTOMATIC PRECLINICAL MODEL[70]	Bersani	2022	SMNΔ7	Antisense oligonucleotide (PMO)	8	P5
A COMBINATORIAL APPROACH INCREASES SMN LEVEL IN SMA MODEL MICE[71]	Dumas	2022	SMNΔ7	Antisense oligonucleotide (PMO)	6	P1
ANTIBODY-OLIGONUCLEOTIDE CONJUGATE ACHIEVES CNS DELIVERY IN ANIMAL MODELS FOR SPINAL MUSCULAR ATROPHY[72]	Hammond	2022	Taiwanese	Antisense oligonucleotide (PMO)	7	P0
COUNTERACTING CHROMATIN EFFECTS OF A SPLICING-CORRECTING ANTISENSE OLIGONUCLEOTIDE IMPROVES ITS THERAPEUTIC EFFICACY IN SPINAL MUSCULAR ATROPHY[73]	Marasco	2022	SMNΔ7	Antisense oligonucleotide (MOE)	14	P0
CENTRAL AND PERIPHERAL DELIVERED AAV9-SMN ARE BOTH EFFICIENT BUT TARGET DIFFERENT PATHOMECHANISMS IN A MOUSE MODEL OF SPINAL MUSCULAR ATROPHY[74]	Reilly	2022	Smn2B/-	Viral vector (AAV9-SMN)	5	P1
ANTISENSE OLIGONUCLEOTIDES TARGETING THE SMN2 PROMOTER REGION ENHANCE SMN2 EXPRESSION IN SPINAL MUSCULAR ATROPHY CELL LINES AND MOUSE MODEL[75]	Wang	2022	SMNΔ7	Antisense oligonucleotide (2'-O-Me)	15	P2
DG9-CONJUGATED MORPHOLINO RESCUES PHENOTYPE IN SMA MICE BY REACHING THE CNS VIA A SUBCUTANEOUS ADMINISTRATION[76]	Aslesh	2023	Taiwanese	Antisense oligonucleotide (MOE; PMO)	4	P0

PRMT INHIBITOR PROMOTES SMN2 EXON 7 INCLUSION AND SYNERGIZES WITH NUSINERSEN TO RESCUE SMA MICE[77]	Kordala	2023	Taiwanese	Antisense oligonucleotide (2'-O-Me)	15	P0
LONG TERM PERIPHERAL AAV9-SMN GENE THERAPY PROMOTES SURVIVAL IN A MOUSE MODEL OF SPINAL MUSCULAR ATROPHY[78]	Reilly	2023	Smn2B/-	Viral vector (AAV9-SMN)	4	P1

References

1. Azzouz M, Le T, Ralph GS, Walmsley L, Monani UR, Lee DCP, et al. Lentivector-mediated SMN replacement in a mouse model of spinal muscular atrophy. J Clin Invest 2004; 114(12).
2. Baughan TD, Dickson A, Osman EY, Lorson CL. Delivery of bifunctional RNAs that target an intronic repressor and increase SMN levels in an animal model of spinal muscular atrophy. Hum Mol Genet 2009; 18(9): 1600–11.
3. Mattis VB, Ebert AD, Fosso MY, Chang CW, Lorson CL. Delivery of a read-through inducing compound, TC007, lessens the severity of a spinal muscular atrophy animal model. Hum Mol Genet 2009; 18(20): 3906–13.
4. Mattis VB, Fosso MY, Chang CW, Lorson CL. Subcutaneous administration of TC007 reduces disease severity in an animal model of SMA. BMC Neurosci 2009; 10: 1–6.
5. Butchbach MER, Singh J, Thorsteinsdóttir M, Saieva L, Slominski E, Thurmond J, et al. Effects of 2,4-diaminoquinazoline derivatives on SMN expression and phenotype in a mouse model for spinal muscular atrophy. Hum Mol Genet 2009; 19(3): 454–67.
6. Coady TH, Lorson CL. Trans-splicing-mediated improvement in a severe mouse model of spinal muscular atrophy. J Neurosci 2010; 30(1): 126–30.
7. Foust KD, Wang X, McGovern VL, Braun L, Bevan AK, Haidet AM, et al. Rescue of the spinal muscular atrophy phenotype in a mouse model by early postnatal delivery of SMN. Nat Biotechnol 2010; 28(3): 271–4.
8. Passini MA, Bu J, Roskelley EM, Richards AM, Sardi SP, O’Riordan CR, et al. CNS-targeted gene therapy improves survival and motor function in a mouse model of spinal muscular atrophy. J Clin Invest 2010; 120(4): 1253–64.
9. Valori CF, Ning K, Wyles M, Mead RJ, Grierson AJ, Shaw PJ, et al. Systemic delivery of scAAV9 expressing SMN prolongs survival in a model of spinal muscular atrophy. Sci Transl Med 2010; 2(35): 1–10.
10. Dominguez E, Marais T, Chatauret N, Benkhelifa-Ziyyat S, Duque S, Ravassard P, et al. Intravenous scAAV9 delivery of a codon-optimized SMN1 sequence rescues SMA mice. Hum Mol Genet 2011; 20(4): 681–93.
11. Hua Y, Sahashi K, Rigo F, Hung G, Horev G, Bennett CF, et al. Peripheral SMN restoration is essential for long-term rescue of a severe spinal muscular atrophy mouse model. Nature 2011; 478(7367): 123–6.
12. Passini MA, Bu J, Richards AM, Kinnecom C, Sardi SP, Stanek LM, et al. Antisense oligonucleotides delivered to the mouse CNS ameliorate symptoms of severe spinal muscular atrophy. Sci Transl Med 2011; 3(72).
13. Shababi M, Glascock J, Lorson CL. Combination of SMN trans-splicing and a neurotrophic factor increases the life span and body mass in a severe model of spinal muscular atrophy. Hum Gene Ther 2011; 22(2): 135–44.
14. Glascock JJ, Shababi M, Wetz MJ, Krogman MM, Lorson CL. Direct central nervous system delivery provides enhanced protection following vector mediated gene replacement in a severe model of Spinal Muscular Atrophy. Biochem Biophys Res Commun 2012; 417(1): 376–81.
15. Glascock JJ, Osman EY, Wetz MJ, Krogman MM, Shababi M, Lorson CL. Decreasing disease severity in symptomatic, Smn-/-;SMN2 ++, spinal muscular atrophy mice following scAAV9-SMN delivery. Hum Gene Ther 2012; 23(3): 330–5.
16. Mattis VB, Tom Chang CW, Lorson CL. Analysis of a read-through promoting compound in a severe mouse model of spinal muscular atrophy. Neurosci Lett 2012; 525(1): 72–5.
17. Osman EY, Yen PF, Lorson CL. Bifunctional RNAs targeting the intronic splicing silencer N1 increase SMN levels and reduce disease severity in an animal model of spinal muscular atrophy. Mol Ther 2012; 20(1): 119–26.

18. Porensky PN, Mitrpant C, McGovern VL, Bevan AK, Foust KD, Kaspar BK, et al. A single administration of morpholino antisense oligomer rescues spinal muscular atrophy in mouse. Hum Mol Genet 2012; 21(7): 1625–38.
19. Shababi M, Habibi J, Ma L, Glascock JJ, Sowers JR, Lorson CL. Partial restoration of cardio-vascular defects in a rescued severe model of spinal muscular atrophy. J Mol Cell Cardiol 2012; 52(5): 1074–82.
20. Benkhelifa-Ziyyat S, Besse A, Roda M, Duque S, Astord S, Carcenac R, et al. Intramuscular scAAV9-SMN injection mediates widespread gene delivery to the spinal cord and decreases disease severity in SMA mice. Mol Ther 2013; 21(2): 282–90.
21. Cherry JJ, Osman EY, Evans MC, Choi S, Xing X, Cuny GD, et al. Enhancement of SMN protein levels in a mouse model of spinal muscular atrophy using novel drug-like compounds. EMBO Mol Med 2013; 5(7): 1103–18.
22. Gogliotti RG, Cardona H, Jasbir S, Bail S, Emery C, Kuntz N, et al. The dcpS inhibitor RG3039 improves survival, function and motor unit pathologies in two SMA mouse models. Hum Mol Genet 2013; 22(20): 4084–101.
23. Mitrpant C, Porensky P, Zhou H, Price L, Muntoni F, Fletcher S, et al. Improved Antisense Oligonucleotide Design to Suppress Aberrant SMN2 Gene Transcript Processing: Towards a Treatment for Spinal Muscular Atrophy. PLoS One 2013; 8(4): 2–11.
24. Zhou H, Janghra N, Mitrpant C, Dickinson RL, Anthony K, Price L, et al. A novel morpholino oligomer targeting ISS-N1 improves rescue of severe spinal muscular atrophy transgenic mice. Hum Gene Ther 2013; 24(3): 331–42.
25. Butchbach MER, Singh J, Gurney ME, Burghes AHM. The effect of diet on the protective action of D156844 observed in spinal muscular atrophy mice. Exp Neurol 2014; 256: 1–6.
26. Keil JM, Seo J, Howell MD, Hsu WH, Singh RN, DiDonato CJ. A short antisense oligonucleotide ameliorates symptoms of severe mouse models of spinal muscular atrophy. Mol Ther Nucleic Acids 2014; 3(7): e174.
27. Naryshkin NA, Weetall M, Dakka A, Narasimhan J, Zhao X, Feng Z, et al. SMN2 splicing modifiers improve motor function and longevity in mice with spinal muscular atrophy. Science (80-) 2014; 345(6197).
28. Nizzardo M, Simone C, Salani S, Ruepp MD, Rizzo F, Ruggieri M, et al. Effect of combined systemic and local morpholino treatment on the spinal muscular atrophy δ7 mouse model phenotype. Clin Ther 2014; 36(3): 340-356.e5.
29. Osman EY, Miller MR, Robbins KL, Lombardi AM, Atkinson AK, Brehm AJ, et al. Morpholino antisense oligonucleotides targeting intronic repressor Element1 improve phenotype in SMA mouse models. Hum Mol Genet 2014; 23(18): 4832–45.
30. Passini MA, Bu J, Richards AM, Treleaven CM, Sullivan JA, O'Riordan CR, et al. Translational fidelity of intrathecal delivery of self-complementary AAV9-survival motor neuron 1 for spinal muscular atrophy. Hum Gene Ther 2014; 25(7): 619–30.
31. Robbins KL, Glascock JJ, Osman EY, Miller MR, Lorson CL. Defining the therapeutic window in a severe animal model of spinal muscular atrophy. Hum Mol Genet 2014; 23(17): 4559–68.
32. Bogdanik LP, Osborne MA, Davis C, Martin WP, Austin A, Rigo F, et al. Systemic, postsymptomatic antisense oligonucleotide rescues motor unit maturation delay in a new mouse model for type II/III spinal muscular atrophy. Proc Natl Acad Sci U S A 2015; 112(43): E5863–72.
33. Hua Y, Liu YH, Sahashi K, Rigo F, Frank Bennett C, Krainer AR. Motor neuron cell-nonautonomous rescue of spinal muscular atrophy phenotypes in mild and severe transgenic mouse models. Genes Dev 2015; 29(3): 288–97.
34. Meyer K, Ferraiuolo L, Schmelzer L, Braun L, McGovern V, Likhite S, et al. Improving single injection CSF delivery of AAV9-mediated gene therapy for SMA: A dose-response study in mice and nonhuman primates. Mol Ther 2015; 23(3): 477–87.

35. Palacino J, Swalley SE, Song C, Cheung AK, Shu L, Zhang X, et al. SMN2 splice modulators enhance U1-pre-mRNA association and rescue SMA mice. Nat Chem Biol 2015; 11(7): 511–7.
36. Rindt H, Feng Z, Mazzasette C, Glascock JJ, Valdivia D, Pyles N, et al. Astrocytes influence the severity of spinal muscular atrophy. Hum Mol Genet 2015; 24(14): 4094–102.
37. Zhou H, Meng J, Marrosu E, Janghra N, Morgan J, Muntoni F. Repeated low doses of morpholino antisense oligomer: An intermediate mouse model of spinal muscular atrophy to explore the window of therapeutic response. Hum Mol Genet 2015; 24(22): 6265–77.
38. Armbruster N, Lattanzi A, Jeavons M, Van Wittenberghe L, Gjata B, Marais T, et al. Efficacy and biodistribution analysis of intracerebroventricular administration of an optimized scAAV9-SMN1 vector in a mouse model of spinal muscular atrophy. Mol Ther - Methods Clin Dev 2016; 3(July): 16060.
39. Arnold WD, McGovern VL, Sanchez B, Li J, Corlett KM, Kolb SJ, et al. The neuromuscular impact of symptomatic SMN restoration in a mouse model of spinal muscular atrophy. Neurobiol Dis 2016; 87: 116–23.
40. Feng Z, Ling KKY, Zhao X, Zhou C, Karp G, Welch EM, et al. Pharmacologically induced mouse model of adult spinal muscular atrophy to evaluate effectiveness of therapeutics after disease onset. Hum Mol Genet 2016; 25(5): 964–75.
41. Hammond SM, Hazell G, Shabanpoor F, Saleh AF, Bowerman M, Sleigh JN, et al. Systemic peptide-mediated oligonucleotide therapy improves long-term survival in spinal muscular atrophy. Proc Natl Acad Sci U S A 2016; 113(39): 10962–7.
42. Hosseinibarkooie S, Peters M, Torres-Benito L, Rastetter RHH, Hupperich K, Hoffmann A, et al. The Power of Human Protective Modifiers: PLS3 and CORO1C Unravel Impaired Endocytosis in Spinal Muscular Atrophy and Rescue SMA Phenotype. Am J Hum Genet 2016; 99(3): 647–65.
43. Lin TL, Chen TH, Hsu YY, Cheng YH, Juang BT, Jong YJ. Selective neuromuscular denervation in Taiwanese severe SMA mouse can be reversed by morpholino antisense oligonucleotides. PLoS One 2016; 11(4): 1–15.
44. Odermatt P, Trüb J, Furrer L, Fricker R, Marti A, Schümperli D. Somatic therapy of a mouse SMA model with a U7 snRNA gene correcting SMN2 splicing. Mol Ther 2016; 24(10): 1797–805.
45. Osman EY, Washington CW, Kaifer KA, Mazzasette C, Patitucci TN, Florea KM, et al. Optimization of morpholino antisense oligonucleotides targeting the intronic repressor element1 in spinal muscular atrophy. Mol Ther 2016; 24(9): 1592–601.
46. Ratni H, Karp GM, Weetall M, Naryshkin NA, Paushkin S V., Chen KS, et al. Specific Correction of Alternative Survival Motor Neuron 2 Splicing by Small Molecules: Discovery of a Potential Novel Medicine to Treat Spinal Muscular Atrophy. J Med Chem 2016; 59(13): 6086–100.
47. Woll MG, Qi H, Turpoff A, Zhang N, Zhang X, Chen G, et al. Discovery and Optimization of Small Molecule Splicing Modifiers of Survival Motor Neuron 2 as a Treatment for Spinal Muscular Atrophy. J Med Chem 2016; 59(13): 6070–85.
48. Zhao X, Feng Z, Ling KKY, Mollin A, Sheedy J, Yeh S, et al. Pharmacokinetics, pharmacodynamics, and efficacy of a small-molecule SMN2 splicing modifier in mouse models of spinal muscular atrophy. Hum Mol Genet 2016; 25(10): 1885–99.
49. d'Ydewalle C, Ramos DM, Pyles NJ, Ng SY, Gorz M, Pilato CM, et al. The Antisense Transcript SMN-AS1 Regulates SMN Expression and Is a Novel Therapeutic Target for Spinal Muscular Atrophy. Neuron 2017; 93(1): 66–79.
50. Kaifer KA, Villalón E, Osman EY, Glascock JJ, Arnold LL, Cornelison DDW, et al. Plastin-3 extends survival and reduces severity in mouse models of spinal muscular atrophy. JCI Insight 2017; 2(5): 1–10.

51. Osman EY, Washington CW, Simon ME, Megiddo D, Greif H, Lorson CL. Analysis of Azithromycin Monohydrate as a Single or a Combinatorial Therapy in a Mouse Model of Severe Spinal Muscular Atrophy. J Neuromuscul Dis 2017; 4(3): 237–49.
52. Shabanpoor F, Hammond SM, Abendroth F, Hazell G, Wood MJA, Gait MJ. Identification of a Peptide for Systemic Brain Delivery of a Morpholino Oligonucleotide in Mouse Models of Spinal Muscular Atrophy. Nucleic Acid Ther 2017; 27(3): 130–43.
53. Gray KM, Kaifer KA, Baillat D, Wen Y, Bonacci TR, Ebert AD, et al. Self-oligomerization regulates stability of survival motor neuron protein isoforms by sequestering an SCF Slmb degron . Mol Biol Cell 2018; 29(2): 96–110.
54. Janzen E, Mendoza-Ferreira N, Hosseinibarkooie S, Schneider S, Hupperich K, Tschanz T, et al. CHP1 reduction ameliorates spinal muscular atrophy pathology by restoring calcineurin activity and endocytosis. Brain 2018; 141(8): 2343–61.
55. Ratni H, Ebeling M, Baird J, Bendels S, Bylund J, Chen KS, et al. Discovery of Risdiplam, a Selective Survival of Motor Neuron-2 (SMN2) Gene Splicing Modifier for the Treatment of Spinal Muscular Atrophy (SMA). J Med Chem 2018; 61(15): 6501–17.
56. Donadon I, Bussani E, Riccardi F, Licastro D, Romano G, Pianigiani G, et al. Rescue of spinal muscular atrophy mouse models with AAV9-Exon-specific U1 snRNA. Nucleic Acids Res 2019; 47(14): 7618–32.
57. Osman EY, Bolding MR, Villalón E, Kaifer KA, Lorson ZC, Tisdale S, et al. Functional characterization of SMN evolution in mouse models of SMA. Scientific Reports 2019; 9(1).
58. Osman EY, Rietz A, Kline RA, Cherry JJ, Hodgetts KJ, Lorson CL, et al. Intraperitoneal delivery of a novel drug-like compound improves disease severity in severe and intermediate mouse models of Spinal Muscular Atrophy. Sci Rep 2019; 9(1): 1–8.
59. Rashnonejad A, Amini Chermahini G, Gündüz C, Onay H, Aykut A, Durmaz B, et al. Fetal Gene Therapy Using a Single Injection of Recombinant AAV9 Rescued SMA Phenotype in Mice. Mol Ther 2019; 27(12): 2123–33.
60. Besse A, Astord S, Marais T, Roda M, Giroux B, Lejeune FX, et al. AAV9-Mediated Expression of SMN Restricted to Neurons Does Not Rescue the Spinal Muscular Atrophy Phenotype in Mice. Mol Ther 2020; 28(8): 1887–901.
61. Ando S, Suzuki S, Okubo S, Ohuchi K, Takahashi K, Nakamura S, et al. Discovery of a CNS penetrant small molecule SMN2 splicing modulator with improved tolerability for spinal muscular atrophy. Sci Rep 2020; 10(1): 1–16.
62. Sheng L, Rigo F, Bennett CF, Krainer AR, Hua Y. Comparison of the efficacy of MOE and PMO modifications of systemic antisense oligonucleotides in a severe SMA mouse model. Nucleic Acids Res 2020; 48(6): 2853–65.
63. Zhou H, Meng J, Malerba A, Catapano F, Sintusek P, Jarmin S, et al. Myostatin inhibition in combination with antisense oligonucleotide therapy improves outcomes in spinal muscular atrophy. J Cachexia Sarcopenia Muscle 2020; 11(3): 768–82.
64. Rietz A, Hodgetts KJ, Lusic H, Quist KM, Osman EY, Lorson CL, et al. Short-duration splice promoting compound enables a tunable mouse model of spinal muscular atrophy. Life Sci Alliance 2021; 4(1).
65. Kong L, Valdivia DO, Simon CM, Hassinan CW, Delestrée N, Ramos DM, et al. Impaired prenatal motor axon development necessitates early therapeutic intervention in severe SMA. Sci Transl Med 2021; 13(578): 1–16.
66. Van Alstyne M, Tattoli I, Delestrée N, Recinos Y, Workman E, Shihabuddin LS, et al. Gain of toxic function by long-term AAV9-mediated SMN overexpression in the sensorimotor circuit. Nat Neurosci 2021; 24(7): 930–40.
67. Hammond SM, Sergeeva O V., Melnikov PA, Goli L, Stoodley J, Zatsepin TS, et al. Mesyl Phosphoramidate Oligonucleotides as Potential Splice-Switching Agents: Impact of Backbone Structure on Activity and Intracellular Localization. Nucleic Acid Ther 2021; 31(3): 190–200.

68. Kray KM, McGovern VL, Chugh D, Arnold WD, Burghes AHM. Dual SMN inducing therapies can rescue survival and motor unit function in symptomatic ∆7SMA mice. Neurobiol Dis. 2021 Nov;159:105488.
69. Zhao X, Feng Z, Risher N, Mollin A, Sheedy J, Ling KKY, et al. SMN protein is required throughout life to prevent spinal muscular atrophy disease progression. Hum Mol Genet. 2021 Dec 17;31(1):82-96.
70. Bersani M, Rizzuti M, Pagliari E, Garbellini M, Saccomanno D, Moulton HM, et al. Cell-penetrating peptide-conjugated Morpholino rescues SMA in a symptomatic preclinical model. Mol Ther. 2022 Mar 2;30(3):1288-1299.
71. Dumas SA, Villalón E, Bergman EM, Wilson KJ, Marugan JJ, Lorson CL, Burnett BG. A combinatorial approach increases SMN level in SMA model mice. Hum Mol Genet. 2022 Aug 25;31(17):2989-3000.
72. Hammond SM, Abendroth F, Goli L, Stoodley J, Burrell M, Thom G, et al. Antibody-oligonucleotide conjugate achieves CNS delivery in animal models for spinal muscular atrophy. JCI Insight. 2022 Dec 22;7(24):e154142.
73. Marasco LE, Dujardin G, Sousa-Luís R, Liu YH, Stigliano JN, Nomakuchi T, et al. Counteracting chromatin effects of a splicing-correcting antisense oligonucleotide improves its therapeutic efficacy in spinal muscular atrophy. Cell. 2022 Jun 9;185(12):2057-2070.e15.
74. Reilly A, Deguise MO, Beauvais A, Yaworski R, Thebault S, Tessier DR, et al. Central and peripheral delivered AAV9-SMN are both efficient but target different pathomechanisms in a mouse model of spinal muscular atrophy. Gene Ther. 2022 Sep;29(9):544-554
75. Wang J, Bai J, OuYang S, Wang H, Jin Y, Peng X, et al. Antisense oligonucleotides targeting the SMN2 promoter region enhance SMN2 expression in spinal muscular atrophy cell lines and mouse model. Hum Mol Genet. 2022 May 19;31(10):1635-1650.
76. Aslesh T, Erkut E, Ren J, Lim KRQ, Woo S, Hatlevig S, et al. DG9-conjugated morpholino rescues phenotype in SMA mice by reaching the CNS via a subcutaneous administration. JCI Insight. 2023 Mar 8;8(5):e160516.
77. Kordala AJ, Stoodley J, Ahlskog N, Hanifi M, Garcia Guerra A, Bhomra A, et al. PRMT inhibitor promotes SMN2 exon 7 inclusion and synergizes with nusinersen to rescue SMA mice. EMBO Mol Med. 2023 Nov 8;15(11):e17683
78. Reilly A, Yaworski R, Beauvais A, Schneider BL, Kothary R. Long term peripheral AAV9-SMN gene therapy promotes survival in a mouse model of spinal muscular atrophy. Hum Mol Genet. 2024 Feb 28;33(6):510-519.
